# Supplementary material for: Spaco: A comprehensive tool for coloring spatial data at single-cell resolution
Source: Patterns (N Y). 2024 Jan 16;5(3):100915. doi: 10.1016/j.patter.2023.100915 (PMC10935509; doi:10.1016/j.patter.2023.100915)
Supplement: Document S1. Figures S1–S6 [file mmc1.pdf]

Patterns, Volume *5*

Supplemental information

Spaco: A comprehensive tool for coloring spatial data at single-cell resolution

Zehua Jing, Qianhua Zhu, Linxuan Li, Yue Xie, Xinchao Wu, Qi Fang, Bolin Yang, Baojun Dai, Xun Xu, Hailin Pan, and Yinqi Bai

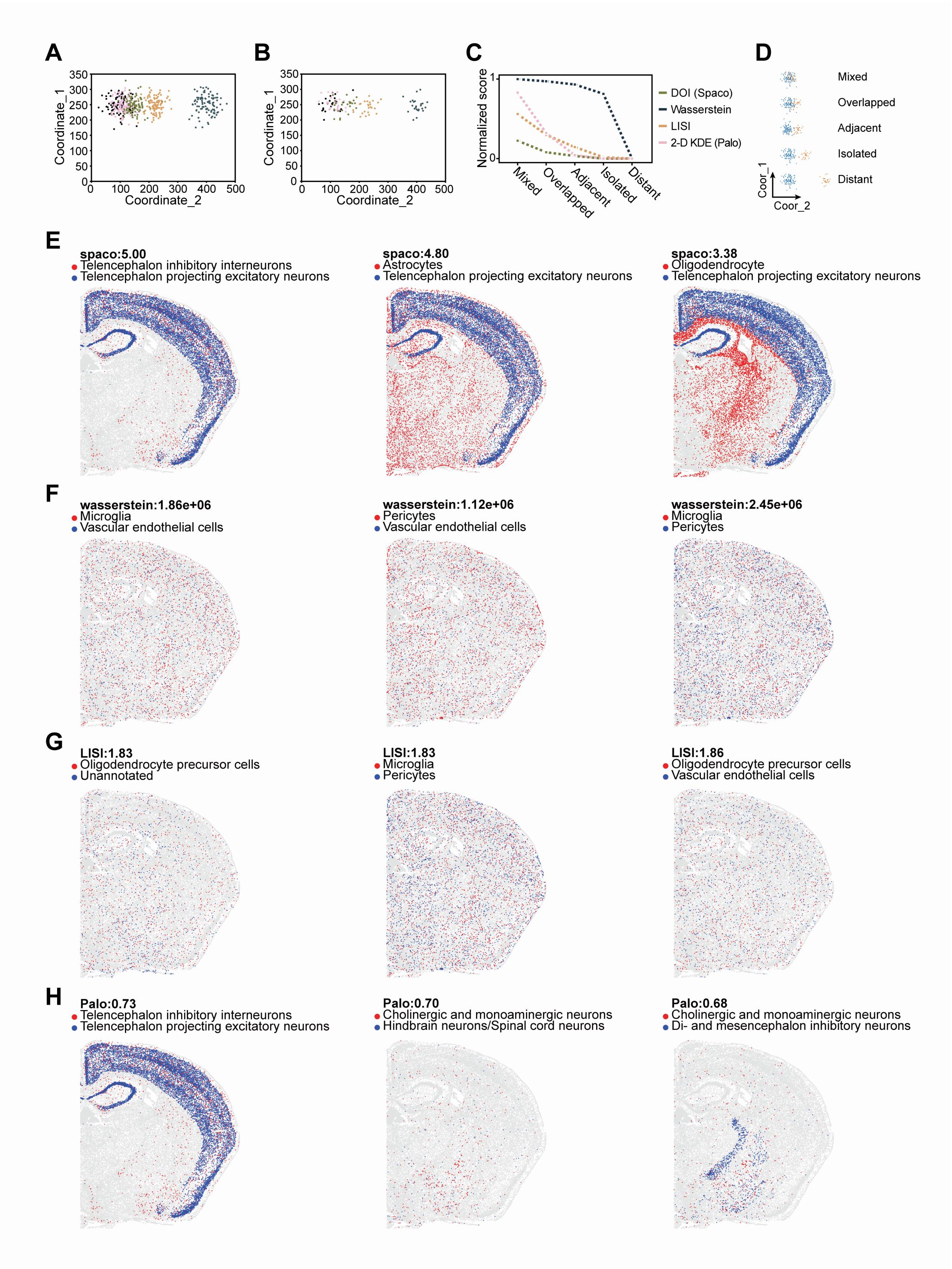

Supplementary Data Figure 1. Evaluation of spatial relation metrics on STARmap mouse brain data

(A) Five groups of points generated to simulate cell type distributions in SRT datasets.

(B) Parallel to panel **A**, with points generated to represent a sparse distribution (see **Methods**).

(C) Min-max normalized scores of five differently distributed cluster pairs in simulation data, evaluated using various metrics. This is akin to **Figure 2B** and **Figure 2C**, but the cluster pairs here are chosen between dense and sparse clusters.

(D) Visualization detailing the spatial distribution of each cluster pair evaluated in panel **C**.

(E-H) Top three scored cluster pairs in the STARmap mouse brain dataset as determined by the DOI metric (**E**), Wasserstein distance (**F**), LISI (**G**) and 2-D KDE-Jaccard index (**H**). The scores and normalized scores (enclosed in brackets) are labeled for each cluster pair and metric.

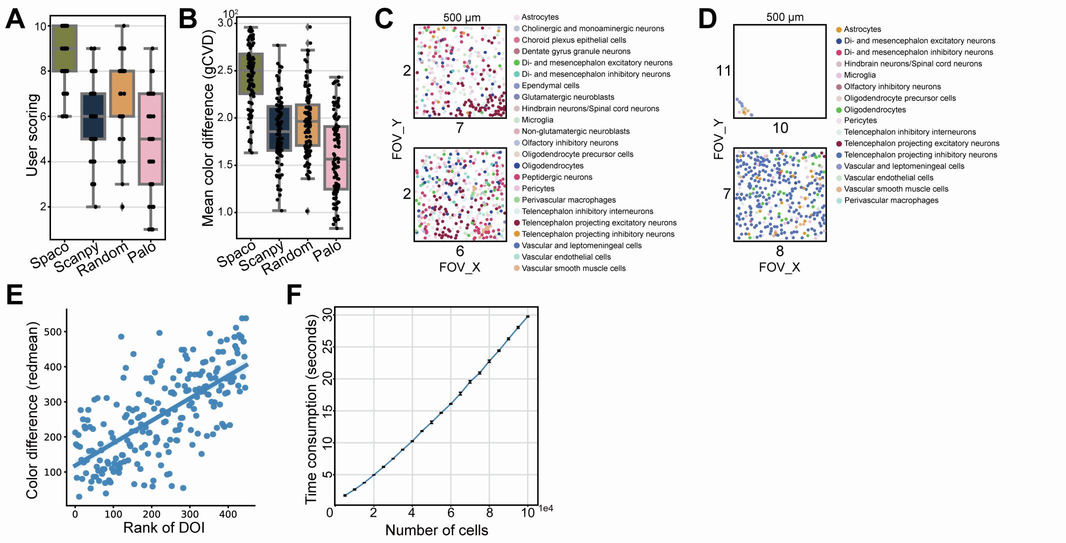

Supplementary Data Figure 2. Evaluation of cluster interlacement scoring and cluster-color matching in Spaco

(A) Blind user scoring of the visualizations from different methods.

(B) Same as **Figure 3F**, but for Spaco's general-CVD support mode.

(C) Same as **Figure 3G**, but for Spaco's general-CVD support mode.

(D) Spaco visualization of the two FOVs with lowest color difference, similar to **Figure 3G**.

(E) Consistency between interlacement scoring and color difference optimized by Spaco, demonstrated by linear regression.

(F) Time consumption of Spaco's color assignment across varying data scales, ranging from 5,000 to 100,000 cells; error bars represent standard deviation.

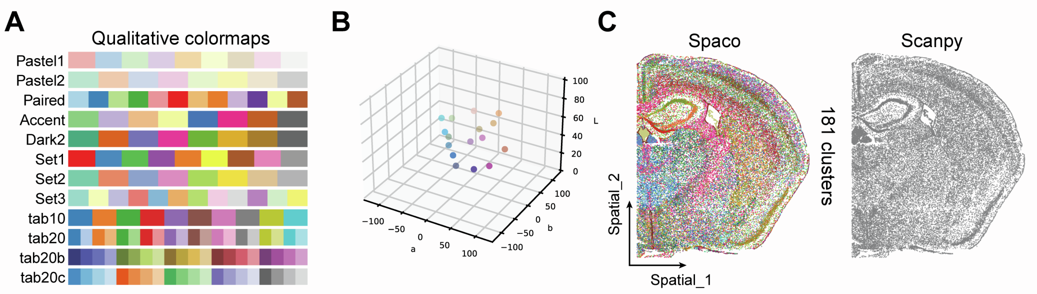

Supplementary Data Figure 3. Evaluation of adaptive graph-guided and image-guided palette selection in Spaco

(A) Commonly used predefined palettes.

(B) Visualization of the colors from the Spaco-generated palette in the 3D CIELab space.

(C) Colorization of 181 cell types annotated in the STARmap dataset using an auto-generated palette by Spaco's cluster interlacement graph embedding (**left**), compared to the glitched palette in Scanpy's default plot (**right**).

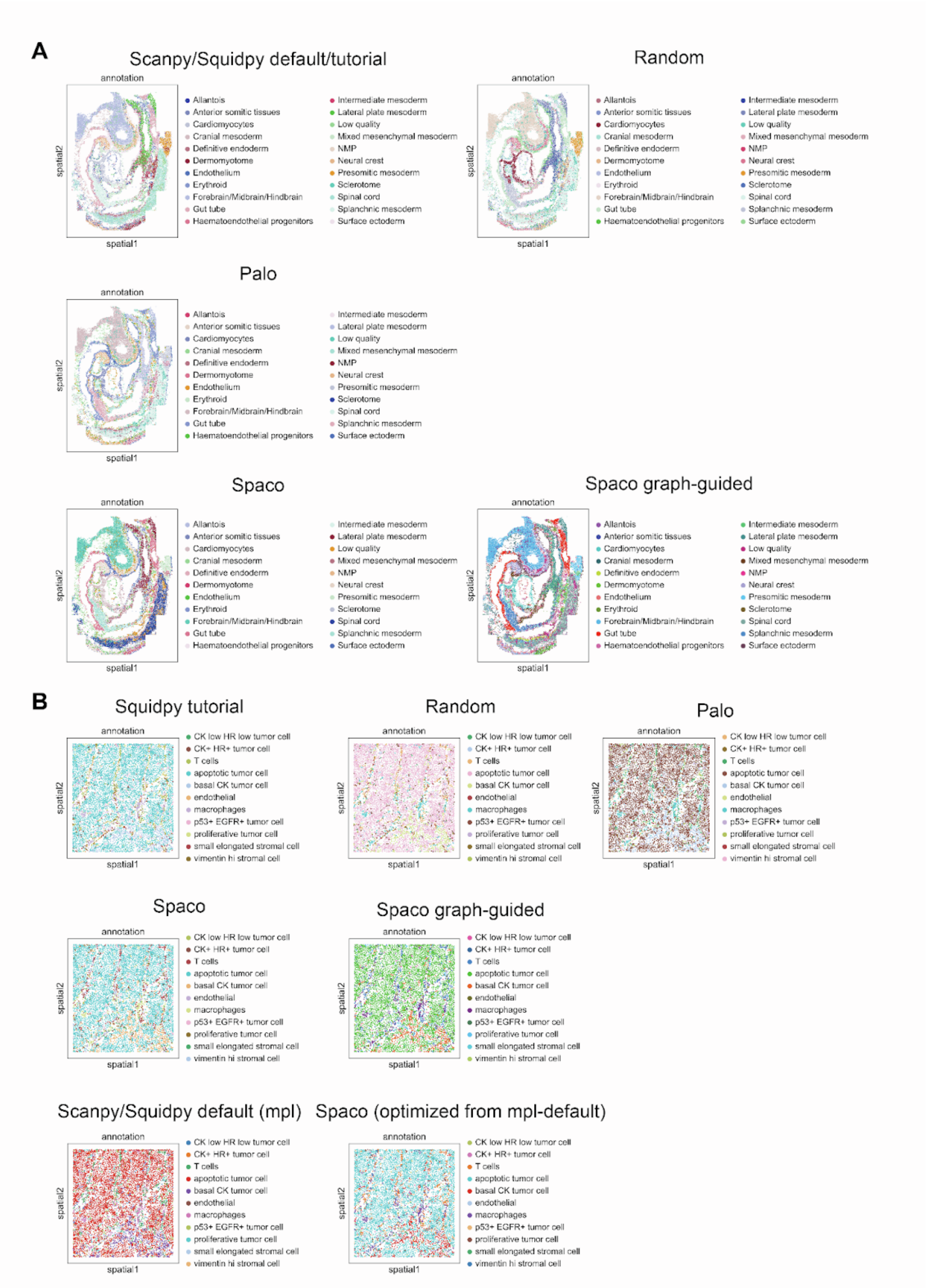

Supplementary Data Figure 4. Benchmarking Spaco on seqFISH and IMC datasets

(A) Colorization of the seqFISH dataset using various colorization strategies. All plots, with the exception of the Spaco graph-guided plot, use the same color palette as the Scanpy/Squidpy default plot, which is also featured in Squidpy tutorials.

(B) Colorization of the IMC dataset using different colorization strategies. The first four plots utilize the color palette from the Squidpy tutorial, while the subsequent plots adopt

the color palette from the Scanpy/Squidpy default plot.

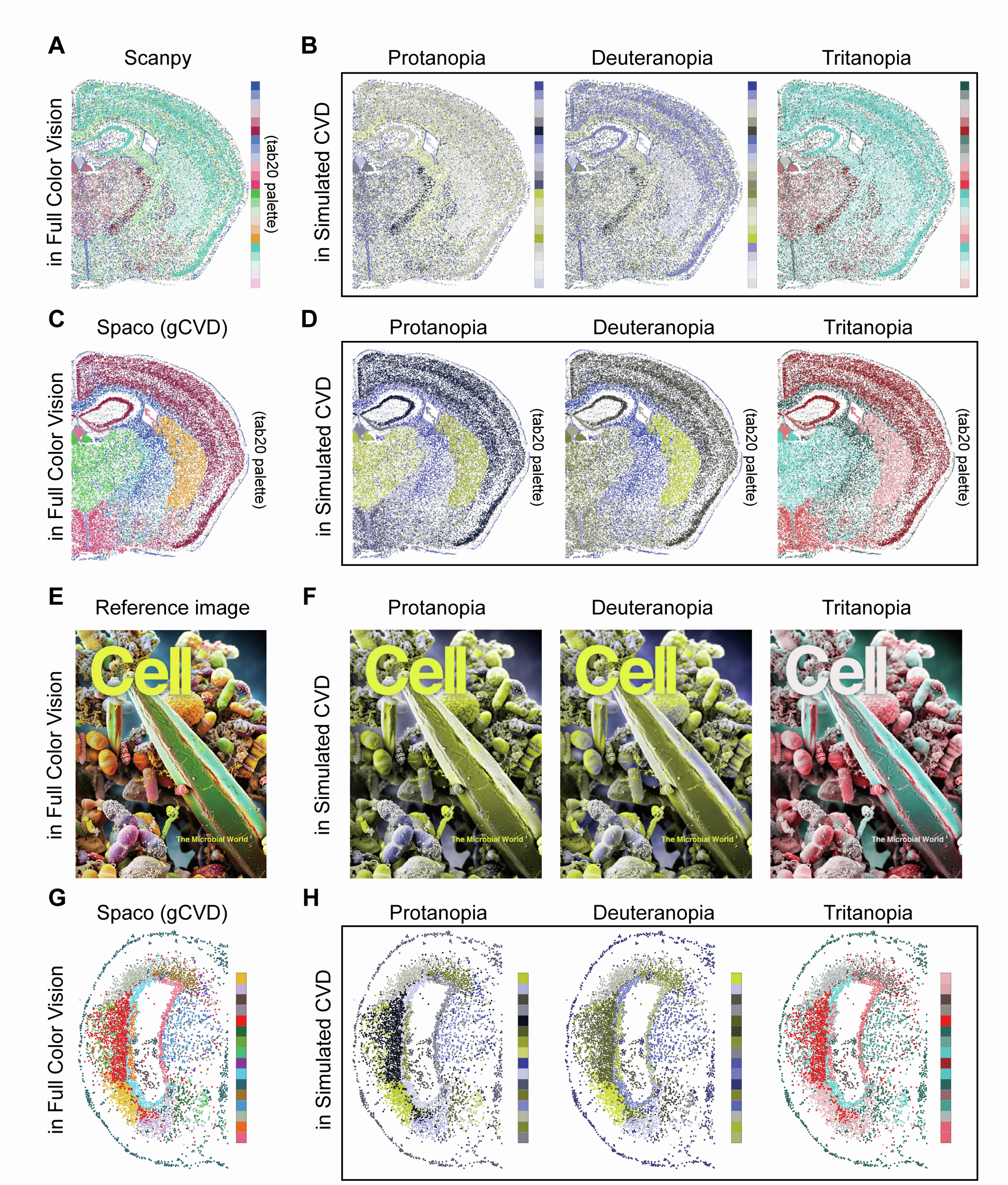

Supplementary Data Figure 5. Testing Spaco's performance for CVD-friendly colorization

(A) Scanpy's default visualization of STARmap mouse brain dataset, demonstrated with full color perception.

(B) Perceptual simulation of different CVDs of Scanpy's default visualization.

(C) Spaco's optimized color assignment of STARmap mouse brain dataset using general-CVD-friendly mode, demonstrated with full color perception.

(D) Same as panel **C**, but plotted with three types of CVD perceptual simulation.

(E) Reference image used in Spaco's palette extraction. Visualized in full color perception.

(F) Same as panel **E**, but visualized in simulated CVD color perception.

(G) Spaco's general-CVD-friendly-mode colorization with image-guided palette extraction on Stereo-seq axolotl brain dataset. Plotted with full color perception.
(H) Same as panel **G**, but plotted to simulate CVD color perception.

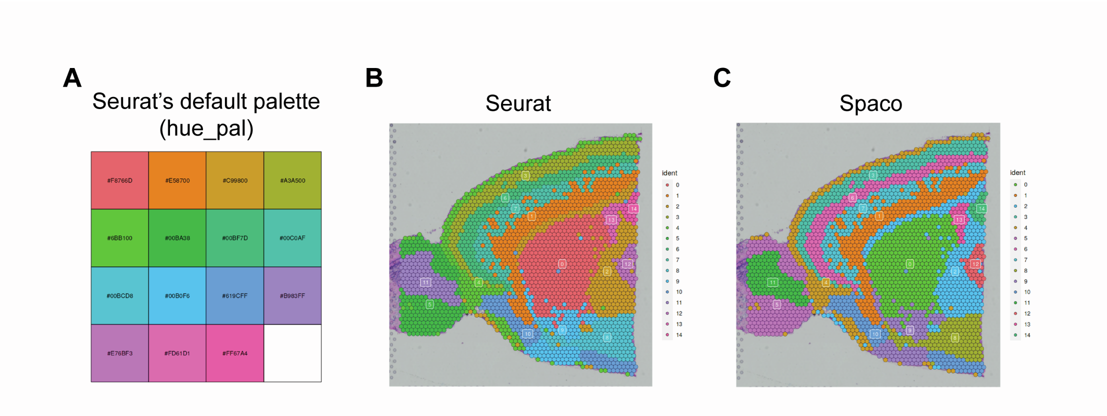

Supplementary Data Figure 6. Demonstration of Spaco’s collaboration with Seurat

(A) Seurat’s default palette selection on 10X Visium mouse brain dataset.

(B) Seurat’s default visualization of 10X Visium mouse brain dataset.

(C) Seurat’s default visualization of 10X Visium mouse brain dataset using optimized cluster-color assignment.
