# Supplementary material for: Spaco: A comprehensive tool for coloring spatial data at single-cell resolution
Source: Patterns (N Y). 2024 Jan 16;5(3):100915. doi: 10.1016/j.patter.2023.100915 (PMC10935509; doi:10.1016/j.patter.2023.100915)
Supplement: Document S2. Article plus supplemental information [file mmc2.pdf]

Patterns

Article

Spaco: A comprehensive tool for coloring spatial data at single-cell resolution

Graphical abstract

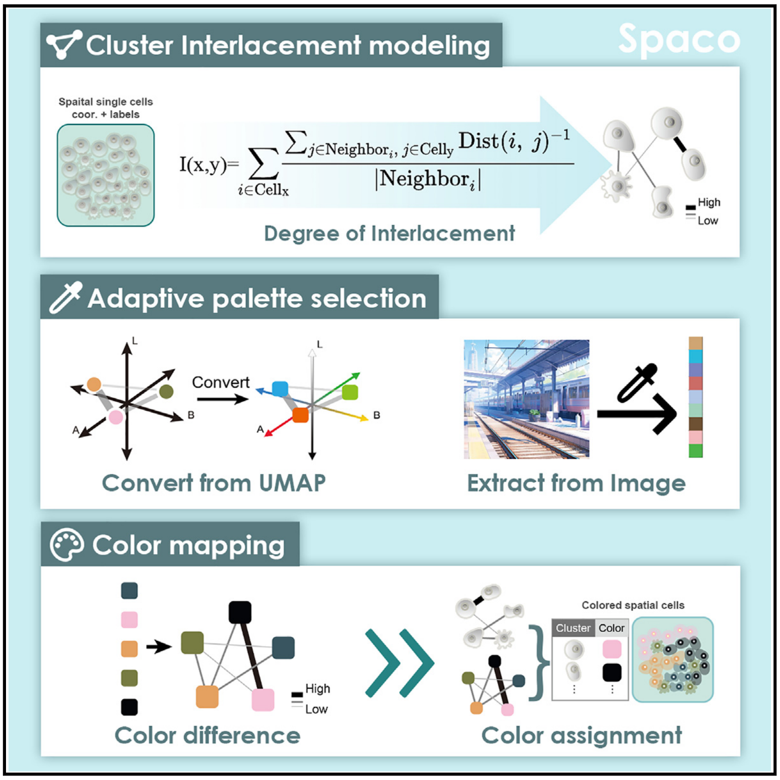

Highlights

- This study underscores the importance of coloring in scientific data visualization
- Authors developed a spatially aware colorization method
- The method proposed a spatial metric modeling the topology among various categories
- Broad accessibility of the method for both full-color vision and colorblind users

Authors

Zehua Jing, Qianhua Zhu, Linxuan Li, ..., Xun Xu, Hailin Pan, Yinqi Bai

Correspondence

xuxun@genomics.cn (X.X.),
panhailin@genomics.cn (H.P.),
baiyinqi@genomics.cn (Y.B.)

In brief

The basic idea to visualize categorical data is to select unique colors for each category and use them to plot labeled data points. However, current colorization strategies often lead to biased visual perception. Jing et al. developed a software to perform spatially aware colorization. The authors proposed a metric to model the neighborship between intricately distributed points and achieved adaptive palette generation with optimized color-category mapping. The method can provide enhanced categorical data visualization in diverse research fields.

Jing et al., 2024, Patterns 5, 100915
March 8, 2024 © 2023 The Author(s).
https://doi.org/10.1016/j.patter.2023.100915

CellPress

Patterns

CellPress
OPEN ACCESS

Article

Spaco: A comprehensive tool for coloring spatial data at single-cell resolution

Zehua Jing,[1,3,6] Qianhua Zhu,[4,6] Linxuan Li,[1,4,6] Yue Xie,[1,4,6] Xinchao Wu,[3,5] Qi Fang,[4] Bolin Yang,[1,3] Baojun Dai,[3,5] Xun Xu,[1,2,4,*] Hailin Pan,[3,4,*] and Yinqi Bai[3,4,7,*]
[1]College of Life Sciences, University of Chinese Academy of Sciences, Beijing 100049, China
[2]Guangdong Provincial Key Laboratory of Genome Read and Write, BGI Research, Shenzhen 518083, China
[3]BGI Research, Hangzhou 310012, China
[4]BGI Research, Shenzhen 518083, China
[5]School of Life Sciences, Southern University of Science and Technology, Shenzhen 518055, China
[6]These authors contributed equally
[7]Lead contact
*Correspondence: xuxun@genomics.cn (X.X.), panhailin@genomics.cn (H.P.), baiyinqi@genomics.cn (Y.B.)
https://doi.org/10.1016/j.patter.2023.100915

THE BIGGER PICTURE Data visualization has become an indispensable step in exploring, interpreting, and deriving insights from large-scale analyses. The use of contrastive colorization to visualize categorical labels can significantly enhance visual perception and aid in uncovering underlying patterns. However, when dealing with spatially resolved data, traditional palettes and lexicographical color-category mappings often result in neighboring categories displaying similar colors, thereby making visual differentiation more difficult. To effectively visualize spatial data, colorization methods must consider the positioning of the data points. Spaco is a space-aware colorization method that can be used across research domains to deal with spatial datasets. This software incorporates a new metric that considers the intricate topology of categorical spatial data and is accessible for both full-color and colorblind users.

1 2 3 4 5 **Proof-of-Concept:** Data science output has been formulated, implemented, and tested for one domain/problem

SUMMARY

Understanding tissue architecture and niche-specific microenvironments in spatially resolved transcriptomics (SRT) requires *in situ* annotation and labeling of cells. Effective spatial visualization of these data demands appropriate colorization of numerous cell types. However, current colorization frameworks often inadequately account for the spatial relationships between cell types. This results in perceptual ambiguity in neighboring cells of biological distinct types, particularly in complex environments such as brain or tumor. To address this, we introduce Spaco, a potent tool for spatially aware colorization. Spaco utilizes the Degree of Interlacement metric to construct a weighted graph that evaluates the spatial relationships among different cell types, refining color assignments. Furthermore, Spaco incorporates an adaptive palette selection approach to amplify chromatic distinctions. When benchmarked on four diverse datasets, Spaco outperforms existing solutions, capturing complex spatial relationships and boosting visual clarity. Spaco ensures broad accessibility by accommodating color vision deficiency and offering open-accessible code in both Python and R.

INTRODUCTION

A comprehensive understanding of tissue architecture is rooted in the exploration of anatomical and histological regions, complemented by the study of cell-type spatial organizations. This pursuit has been profoundly advanced by the emergence of spatially resolved transcriptomics (SRT),[1–5] which has illuminated recent studies in neuroscience,[6,7] developmental biology,[8,9] and a range of diseases.[10,11] To visualize and explore these datasets, cells are *in situ* annotated and labeled, creating a

This is an open access article under the CC BY license (http://creativecommons.org/licenses/by/4.0/).

cellular cartography where each cell type is distinguished by a unique color.[12] Therefore, colorization—the process of selecting and assigning colors[13] to spatially intricately distributed cell clusters—is pivotal to ensure unbiased visual perception and facilitate downstream biological analyses.

Currently, popular data analysis frameworks like Seurat,[14] Giotto,[15] Scanpy,[16] and Squidpy[17] derive their colorization approaches from widely used data visualization libraries, such as matplotlib[18] in Python or ggplot2[19] in R. These methods typically allocate a color palette to cell types either alphabetically or based on descending cell count per type unless a specific order has been manually set. Given that these colorizing principles do not consider the spatial positions of cells, neighboring cells of distinct types often exhibit strikingly similar colors. This becomes particularly challenging for clear visualization in niche-specific microenvironments teeming with multiple cell types, exemplified by brain tissues or tumor niches.

The colorization of cell types for SRT data can be defined as a mathematical problem: given a limited palette, how can we assign more contrasting colors to adjacent cell-type pairs? This poses the challenge of determining the overall proximity of one cell type, which may comprise thousands of cells and is intricately distributed across space, to another. In practice, two cell types might be isolated, adjacent, partially interlaced, or entirely intertwined. However, current public metrics only capture some of these spatial relationships. For instance, the Wasserstein distance[20] measures the degree of isolation between non-overlapping cell types but lacks sensitivity to various interlaced scenarios. In contrast, the local inverse Simpson's index[21] is used to quantify local diversity, determining if cell types are thoroughly intermixed. Palo, a recent palette optimization method,[22] employs the Jaccard index to measure overlaps between the spatial territories of two cell types but shows bias toward spatially sparse cell types.

On the other hand, most existing visualization methods employ pre-set palettes from sources like matplotlib[18] or colorbrewer.[23] While these palettes are visually appealing, they eschew high-contrast colors, thereby reducing discernibility. This becomes pressing when coloring high-resolution SRT datasets containing hundreds of cell types. In addition, these palettes did not support color vision deficiency (CVD).[13]

To address these challenges, we developed Spaco, a comprehensive tool tailored for spatially aware colorization of cellular-resolution SRT data. Spaco introduces the Degree of Interlacement (DOI) metric to evaluate the spatial relationship between individual cell-type pairs. Subsequently, a weighted graph, referred to as the cluster interlacement graph (CI-graph), is constructed to globally model these relationships. Using the CI-graph, Spaco facilitates spatially aware cell-color assignment, ensuring that cluster pairs with larger DOIs are visualized in more distinct colors. For enhanced palette discernibility, Spaco introduces an automatic, spatially aware palette selection approach that exploits the embedding of CI-graph vertices to optimally utilize the visible color spectrum. Furthermore, Spaco shows its capabilities to integratively apply these colorization strategies across multiple tissue sections, aiding spatial differential analyses. It can also derive theme color palettes from high-quality images, making it easier to attain aesthetically pleasing, publication-ready colors. Notably, Spaco ensures its broad accessibility by providing CVD support and public distributed codes in both Python and R.

We benchmarked Spaco on four real-world datasets from diverse cellular-resolution SRT assays and tissue types. These datasets include a STARmap mouse brain dataset,[24] a Stereo-seq axolotl brain dataset,[25] a sequential fluorescence *in situ* hybridization (seqFISH) mouse embryo dataset,[9] and an imaging mass cytometry (IMC) human breast cancer dataset.[26] Our findings illustrate Spaco's capability to capture a variety of spatial relationships using the DOI metric, produce cell-color assignments with enhanced visual clarity, and curate palettes with hundreds of colors to distinguish fine-grained cell types. Furthermore, Spaco significantly aids downstream biological research, such as detailing intricate anatomical structures and cell co-localization in the mouse brain or highlighting the spatial proliferation of specific cell types in wound-healing axolotls. In summary, our results indicate that Spaco offers superior visual clarity for SRT data visualization compared to existing methodologies.

RESULTS

Spaco basics and workflow

Generally, the Spaco colorization process is composed of three primary modules: (1) cell cluster interlacement modeling, (2) color palette selection, and (3) cluster-color assignment (Figure 1A).

In the first module, Spaco accepts a spatial transcriptomic dataset with cell coordinates and corresponding cell-type labels as input. Then, Spaco establishes a DOI metric to form the CI-graph that models the overall topology between clusters, where vertices represent different cell clusters and each edge represents the DOI-assessed spatial relationship between two cell clusters. The DOI is computed via a modified spatial k-nearest neighbor network incorporating a dual-outlier-free strategy, which specifically excludes both spatially sparse cells and cell types in its calculation to enhance the stability (Figure 1B; see the experimental procedures).

The subsequent module focuses on selecting a discernible color palette and calculating the perceptual differences between selected colors with optional CVD support. In color selection, users have the option to choose from two modes. The first and default mode is to automatically generate colors based on pre-calculated CI-graph (Figure 1C; see experimental procedures). Spaco computes the 3D embedding of the CI-graph vertices using the uniform manifold approximation and projection (UMAP) algorithm.[28] As these vertex embeddings are expected to retain their relative distances from the original graph, we then scale them to the perceptually uniform CIELab color space,[29] allowing each vertex to acquire a Lab color. This results in a palette that optimally utilizes the entire color space. The alternative mode does not use spatial information but is designed to facilitate the acquisition of aesthetic colors for publications (Figure 1D; see experimental procedures). Given a user-preferred image, Spaco assesses the color frequencies within the image. It then extracts colors iteratively in descending order of these frequencies and updates the frequencies after each iteration to avoid repetitive extraction of similar colors. The iteration stops when the needed number of colors is extracted, forming an initial color palette. The discernibility of the initial palette is further

CellPress
OPEN ACCESS

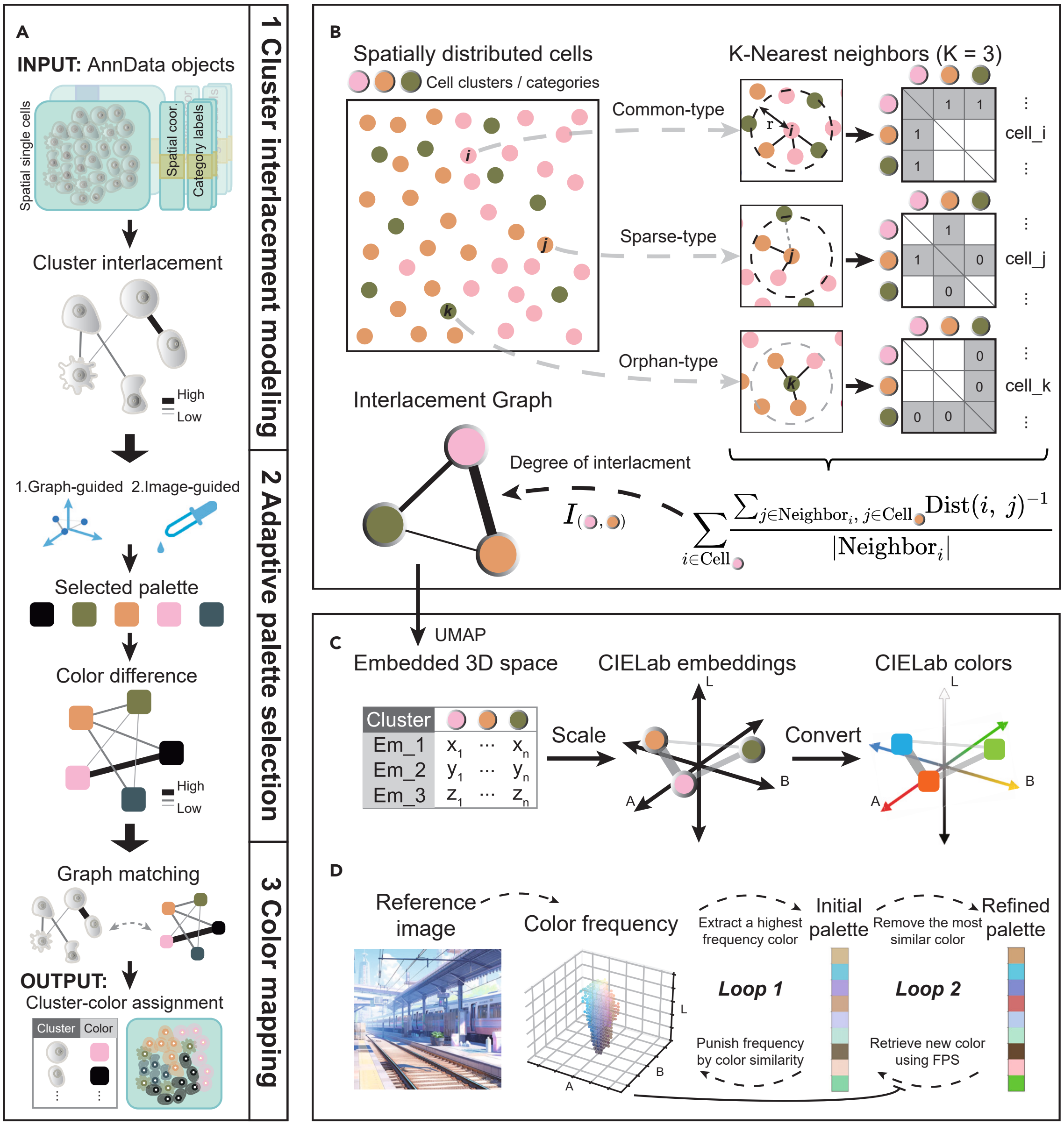

Figure 1. Framework of Spaco for colorization of single-cell-resolution spatial data

Spaco accepts the coordinates of spatial single cells (or cell centroids) along with the categorical (i.e., cell-type) label of each cell, which can be provided in the widely used AnnData format.[27] The colorization process consists of three main parts (A). The first part, referred to as cluster interlacement modeling, employs a dual-outlier-free spatial nearest-neighbor algorithm (B) to calculate the Degree of Interlacement (DOI) between cell clusters, forming a cluster interlacement graph (CI-graph). The second part (adaptive palette selection) offers users two modes for color palette selection: a graph-guided algorithm for automatic palette selection based on a previously calculated CI-graph (C) and an image-guided algorithm that extracts an aesthetic theme palette from a user-provided image (D). Once the palette is selected, perceptual differences between all colors are calculated to construct a color difference graph (CD-graph). The final part utilizes a coarse-to-fine stochastic optimization to match the two graphs, resulting in a cluster-color assignment where heavily interlaced clusters are assigned colors with high perceptual differences (see experimental procedures).

refined using farthest point sampling (FPS).[30] Both modes allow manual adjustments by users, and the image-palette extraction is supplemented with optional CVD support. After the palette selection, Spaco computes a color difference graph (CD-graph), where the vertices represent the selected colors and the edges denote pairwise perceptual differences with optional CVD simulation (see experimental procedures).

In the final module, Spaco delegates cluster-color assignments (i.e., pairing) by identifying an optimal match between the CI-graph and the CD-graph (see experimental procedures). A coarse-to-fine stochastic optimization is used to determine these assignments. This graph-matching process aims to find an optimal correspondence (i.e., one-to-one relationship) between the vertices of the CI-graph and those of the CD-graph such

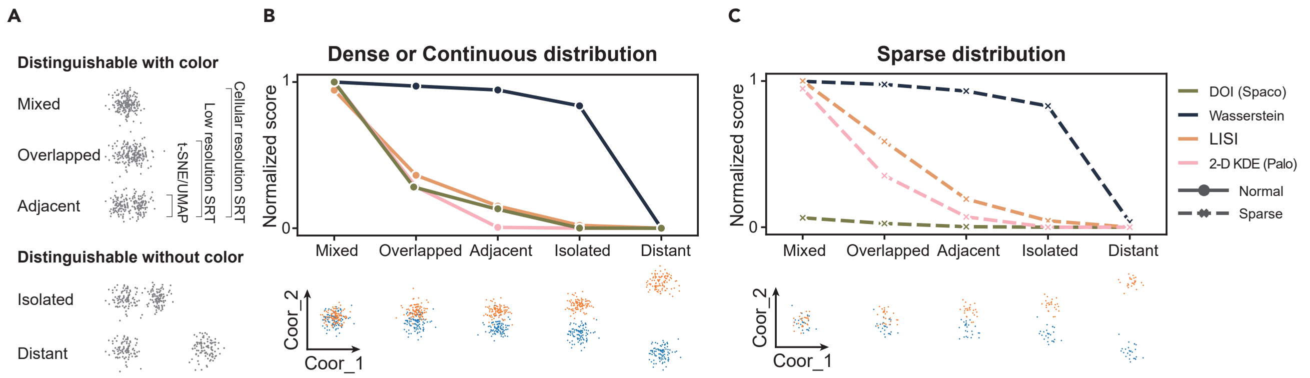

Figure 2. Benchmarking the DOI metric using simulated data

(A) Illustration of five typical spatial relationships observed in cellular SRT data. Colored sidebars highlight the occurrence of these relationships in UMAP-like (single-cell transcriptomic) visualizations, low-resolution SRT, and cellular-resolution SRT data.

(B) Min-max normalized scores for five differently distributed cluster pairs in the simulation data, evaluated using various metrics (top), accompanied by a visualization detailing the spatial distribution of each cluster pair (bottom).

(C) Parallel to (B) but for cluster pairs simulated with a sparse constraint (see experimental procedures).

that the differences between the edges of the two graphs are minimized. Spaco interprets the correspondence between the CI-graph and CD-graph vertices as an optimized color assignment for cell clusters. The minimized difference between the corresponding edges of the CI- and CD-graphs ensures that clusters with larger DOIs are paired with more distinct colors.

Evaluation and benchmarking of DOI metric

Measuring the spatial relationships of cell clusters is fundamental to spatially aware colorization. To assess DOI's capability in measuring interlacement, we first tested it on simulated data. We generated 10 sets of 2D points, with each set simulating the spatial coordinates of a unique cell cluster (Figures S1A and S1B; see experimental procedures). These clusters were arranged to represent five typical types of spatial relationships: completely mixed, partially interlaced, adjacent, isolated, and distantly isolated (Figure 2A). Notably, the primary goal of colorization is to enhance the visual distinction among mixed, overlapped, or adjacent clusters. Therefore, we examined the DOI's capability to differentiate these three scenarios from the other two (isolated and distant). We benchmarked it against three typical types of spatial relationship metrics: distance-based metrics, entropy-based metrics, and spatial-coverage-based metrics. These were exemplified by the Wasserstein distance, the local inverse Simpson's index (LISI), and the 2D kernel density estimation-Jaccard index (2D KDE), respectively.

When cells display a dense or continuous distribution, all metrics recorded the highest scores for the mixed cluster pair and the lowest for the distantly isolated pair (Figure 2B). However, the Wasserstein distance showed a significant difference between the isolated pair and the distantly isolated pair due to its sensitivity to spatial distance. The 2D KDE scored zero for all three non-overlapping pairs, failing to identify the adjacent pair. Both the DOI and LISI successfully distinguished the two isolated cases from the non-isolated ones, serving as appropriate spatial relation metrics to indicate the necessity for distinct colors.

On the other hand, within a real tissue context, certain cell types, such as immunocytes, may display a relatively sparse distribution compared to others. While these cells typically do not dominate the global visualization of an SRT section, they play a crucial role in the biological exploration of cellular niches (e.g., the tumor-immune microenvironments). The DOI metric captured the neighborship (whether mixed, overlapped, or adjacent) of these sparse cluster pairs with relatively low scores. In contrast, other metrics did not demonstrate significant sensitivity to cell sparsity (Figures 2C, S1C, and S1D). This suggests that the dual-outlier-free strategy employed in the DOI calculation can prevent sparse clusters from having a disproportionate impact during the colorization process.

The robustness of the DOI in response to varying cell sparsity was further illustrated using a public STARmap mouse brain dataset.[24] We presented the cluster pairs with the top three highest scores across all metrics (Figures S1E and S1H). The DOI identified the mixture of inhibitory and excitatory neurons, the partial interlacement between astrocytes and neurons, and the adjacency between the cortex and fiber tract. These observations align with widely discussed topics in cell co-location and brain structure studies[31] (Figure S1E). The Wasserstein distance and LISI seemed to favor cell types with globally similar distributions (Figures S1F and S1G). While these globally sparsely distributed cells might have significant roles in brain functions, their spatial relationships offer limited insight into tissue heterogeneity. The 2D KDE echoed the DOI by emphasizing the mixed neuron subtypes in the cortex (Figure S1H). However, it overestimated the spatial neighborship of sparsely co-located cell types, as its kernel density estimation had a tendency to exaggerate the spatial coverage of sparsely distributed cells.

Collectively, these results demonstrate the DOI's capability to capture spatial neighborships with biological significance and to serve as a proper metric to constrain the colorization of SRT data.

Spaco facilitates the exploration of anatomic structure and cell heterogeneity

Next, we evaluated Spaco's cell-color assignment method. In high-definition spatial transcriptomics, effective colorization should cater to both a global overview within a large field of

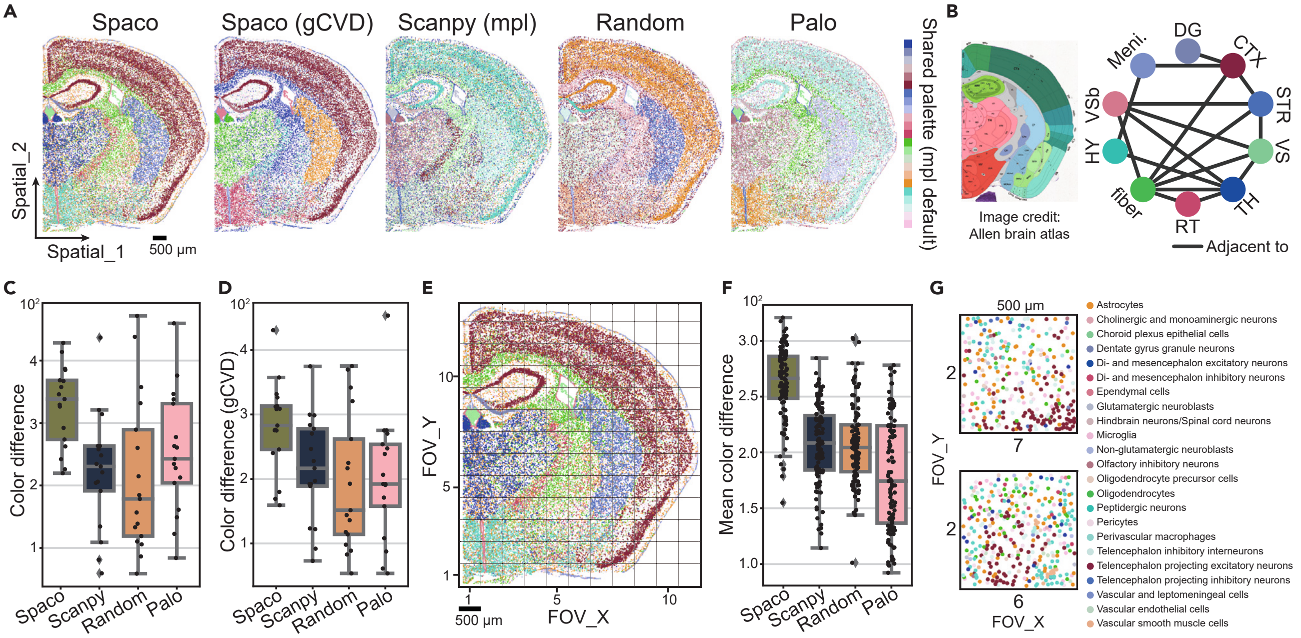

Figure 3. Benchmarking Spaco color assignment on STARmap mouse brain data

(A) Color assignment of 23 brain-related cell subtypes using Spaco, Spaco's general-CVD-support mode, Scanpy (matplotlib), random shuffling, and Palo. All methods shared the same color palette generated from Scanpy (matplotlib) by default.

(B) Anatomical reference from the Allen Brain Atlas[31] corresponding to the STARmap mouse brain section (left) and neighborship between cell clusters that are enriched in one of the brain areas determined according to the anatomical map (right). The vertices of the graph are colored the same as in (A).

(C) Benchmarking of anatomical visualization in Spaco against other methods. Scatters represent the color difference (see experimental procedures) between anatomically adjacent cluster pairs determined in (B).

(D) Same as (C) but for Spaco's general-CVD-support mode.

(E) Gridded fields of view (FOVs) of the mouse brain section with 500 × 500 μm each.

(F) Benchmarking of cellular visualization in Spaco against other methods. Scatters represent the mean cell-cell color difference within each FOV in (E).

(G) Spaco visualization of the two FOVs with the highest color difference.

view (FOV) and detailed perspectives of micro-FOVs. This dual capacity is biologically relevant for studying anatomical tissue structures and discerning niche heterogeneity. To showcase Spaco's visualization proficiencies in these contexts, we applied our method to the STARmap mouse brain dataset,[24] which includes 23 pre-annotated cell clusters. These clusters display either spatially dispersed or aggregated distributions, offering a comprehensive assessment of Spaco's color assignment algorithm (Figure 3A).

We compared Spaco's result with those of Scanpy[16] (utilizing the default matplotlib visualization), random-shuffled assignment, and Palo[22] (Figure 3A), all using the same color palette. To benchmark performance based on brain anatomical features, we identified 10 anatomic-region-localized cell clusters (predominant in one anatomic region) after mapping cell types to the Allen Brain Atlas,[31] yielding 17 spatially adjacent cluster pairs (Figure 3B). Our analysis revealed that Spaco achieved significant visual differences between these anatomically adjacent pairs, as determined by one-sided Wilcoxon tests (no-CVD-support mode, $p = 1.45e{-}4$, $2.32e{-}3$, and $4.51e{-}3$ when compared to Scanpy, random assignments, and Palo, respectively) (Figures 3C and 3D; see experimental procedures). Additionally, we distributed a questionnaire to experts, comprising 19 neurologists and 34 bioinformaticians, asking them to grade each colorization outcome on a scale of 1–10 based on their professional experience. This survey further underscored the superior visual effectiveness of Spaco's colorization approach (Figure S2A; see experimental procedures).

On the other hand, exploring microenvironment heterogeneity requires the zoomed-in visualization of cellular niches. To simulate such scenarios, we arbitrarily divided the data into micro-FOVs measuring 500 × 500 μm each and scored the discernibility of each FOV based on the mean of pairwise color differences among all present cells (Figure 3E; see experimental procedures). The overall discernibility scores across all FOVs were significantly higher in Spaco's visualizations, as shown by one-sided Wilcoxon tests (no-CVD-support mode, $p = 8.42e{-}21$, $9.16e{-}19$, and $8.87e{-}21$ when compared to Scanpy, random assignments, and Palo, respectively) (Figures 3F and S2B). FOVs with the highest discernibility scores in Spaco highlighted the co-localization of various neuron subtypes, such as glutamatergic (excitatory), GABAergic (inhibitory), and peptidergic neurons, around the amygdalar area (Figures 3G and S2C). In contrast, the FOVs with the lowest scores were typically situated at tissue boundaries or contained fewer clusters, indicating that they were less valuable for analyzing cellular heterogeneity (Figure S2D).

Additionally, we sought to determine whether our CI-graph and DOIs contributed to Spaco's enhanced performance. A notable correlation between DOI scores and color differences

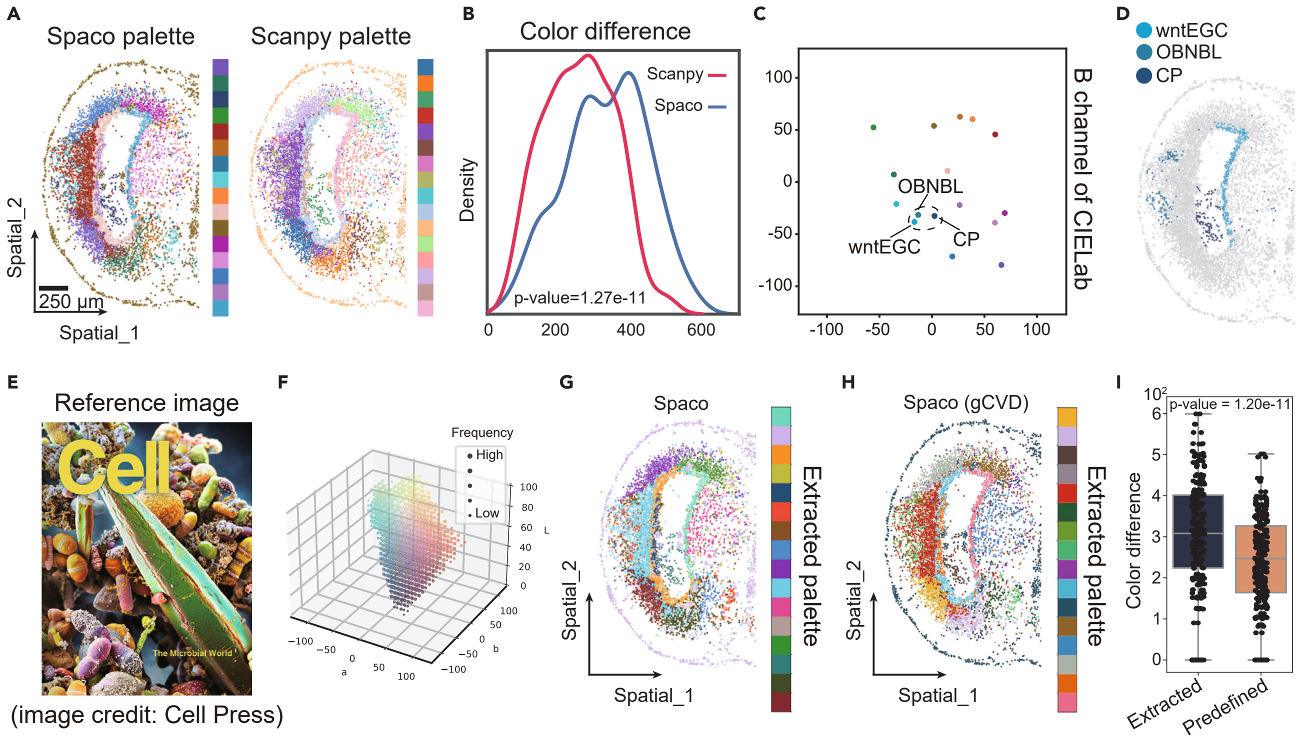

Figure 4. Evaluation of adaptive graph-guided and image-guided palette selection in Spaco

(A) Colorization of 16 cell clusters in the Stereo-seq axolotl brain dataset,[25] using palettes generated by Spaco cluster interlacement graph embedding (left) and Scanpy's default palette (right).

(B) Distribution of pairwise color differences within the Spaco-generated palette in (A), compared to Scanpy's default palette.

(C) Visualization of the colors from the Spaco-generated palette in the 2D CIELab space (channels a and b).

(D) Spatial distributions of cell clusters that are highlighted in (C).

(E) Public journal cover image used to create the image-guided palette (sourced from *Cell*, March 08, 2018, volume 172, issue 6).

(F) Colors and their frequencies derived from the reference image in (E).

(G) Colorization of 16 cell clusters in the Stereo-seq axolotl brain dataset using the palette extracted from the image in (E).

(H) Same as (G) but using Spaco's general-CVD-support mode.

(I) Pairwise color differences within the palette extracted from the image compared to Scanpy's default palette.

(linear regression R-square = 0.45, p = 4e−61) indicated that the subsequent optimization effectively utilized the DOI in Spaco's color-mapping process (Figure S2E). We further evaluated the time complexity of our algorithm. The colorization process required less than 30 s for simulated datasets encompassing up to 100,000 cells (Figure S2F). Hence, the additional computations do not introduce a substantial time burden.

Spaco improves palette discernibility by adaptive color selection

Above, while Spaco effectively enhanced visualization by optimizing the cell-color assignment, its overall performance was still constrained by the discernibility of pre-defined palettes[18,19,23] (Figure S3A). Therefore, we extended the efficacy of Spaco's adaptive color selection algorithm to augment palette discernibility and evaluated its two modes of color selection separately (see experimental procedures).

Firstly, we applied our CI-graph-guided color selection approach to a published Stereo-seq dataset of wound-healing axolotl brain,[25] which contains 16 pre-annotated cell types (Figure 4A). We found that the discernibility of the adaptively generated palette, as quantified by the distribution of pairwise color differences among all palette colors, was significantly higher than that of the pre-defined palette (one-sided Wilcoxon test, p = 1.27e−11) (Figure 4B). Furthermore, the Spaco-generated palette displayed a bimodal distribution of color differences, suggesting an imbalanced color selection across the color space. This indicates that Spaco adeptly integrated the biases of DOIs in the CI-graph, tailoring color selection to accommodate both distant and adjacent cluster pairs (Figures 4C and S3B). Analyzing these colors in the CIELab space, we pinpointed three neighboring cell types associated with shades of blue. These colors corresponded to the embedding of spatially distant cell clusters with low DOIs, which are easily distinguishable through subtle color variations (Figure 4D). Leveraging this, Spaco also effectively crafted a suitable palette for the clear visualization of the STARmap dataset comprising 181 fine-grained cell types (Figure S3C). In contrast, matplotlib failed to capture such a plethora of clusters, producing an uncolored plot due to its limited color pre-sets.

While maximizing color differences can enhance daily data visualization, researchers occasionally prefer to adopt coloring

CellPress
OPEN ACCESS

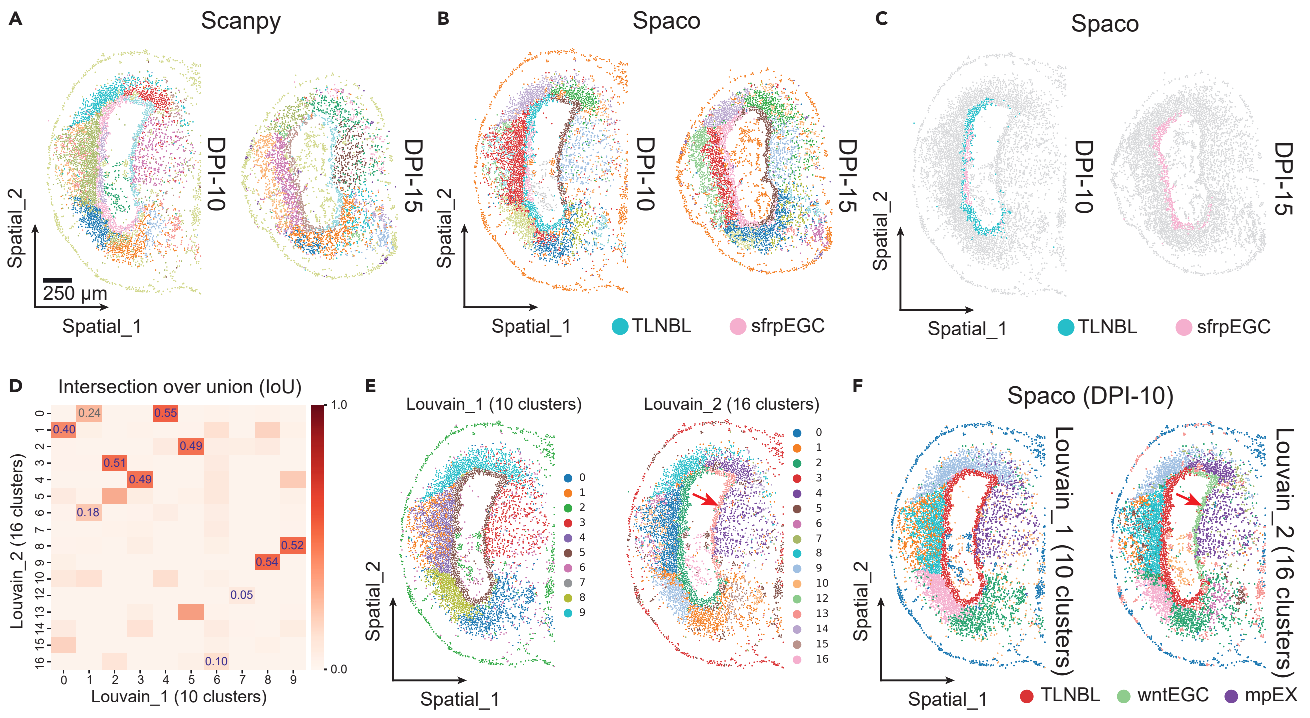

Figure 5. Integrative colorization across multiple plots by Spaco

(A) Scanpy colorization of two Stereo-seq axolotl datasets (days post-injury 10 and 15), with separate color assignment by default.

(B) Spaco colorization of the Stereo-seq axolotl datasets using integrative color assignment and sharing the same palette as the Scanpy default plots.

(C) Highlighted spatial distribution of telencephalon neuroblasts (TLNBLs) and Sfrp+ ependymoglial cells (sfrpEGCs) in (B).

(D) Cluster-wise intersection over union (IoU) score between two Louvain clustering results; top IoU scores are labeled. Blue digits represent the final correspondences derived using Spaco's greedy strategy (see experimental procedures).

(E) Scanpy colorization of two different Louvain clustering results of DPI-10 dataset, using separate color assignments by default. Red arrow highlights the TLNBL cell cluster.

(F) Spaco colorization of the two different Louvain clustering results of DPI-10 dataset, using aligned cluster label from (D) and an integrative color assignment and palette selection based on the cluster interlacement graph. Red arrow highlights the TLNBL cell cluster.

themes from prior publications or presentations. Spaco supports this requirement with its image-guided palette extraction mode (see experimental procedures). We illustrated this process using a public journal cover image as shown in Figure 4E. The extracted colors, and their subsequent application to the axolotl dataset, showed comparable discernibility to a predefined color palette (tab20 in matplotlib colormap) (Wilcoxon test, $p = 1.20e-11$) (Figures 4F–4I). Importantly, this was achieved while preserving the thematic consistency of the original image.

These results underscore Spaco's capability to navigate the confines of the limited visible color space, achieving superior perceptual discernibility in both daily and publication scenarios.

Spaco integratively colors cell clusters across multiple SRT sections

A subsequent challenge in SRT data analysis lies in discerning variations in spatial cell distribution across diverse biological samples. This requires the visualization of multiple tissue sections using a uniform palette and color assignment scheme. To address this, Spaco has been enhanced with a feature that facilitates integrative colorization by seamlessly merging CI-graphs (see experimental procedures).

We applied Spaco to a time-series study of axolotl brain regeneration.[25] The optimized visualization highlighted an increase in Sfrp+ ependymoglial cells (sfrpEGCs) during the wound-healing progression from 10 days post-injury (DPI-10) to DPI-15. This increase was difficult to detect using Scanpy's default visualization due to challenges from incorrectly matching telencephalon neuroblasts (TLNBLs) at DPI-10 with sfrpEGCs at DPI-15 (Figures 5A–5C).

Additionally, we employed a method to streamline the tuning process in clustering analysis. This approach involved comparing clustering outcomes generated using different parameters. By computing Jaccard indices and employing a greedy matching algorithm, we established correspondences between clusters (Figure 5D; see experimental procedures). Contrary to Louvain's default colorization method, which entirely altered the colors between the two rounds clustering of the same dataset (Figure 5E), Spaco retained the colors for the cell types annotated in the first round. It then suitably designated colors to newly emerged cell subtypes *in situ*. This directly distinguished WNT+ EGCs (wntEGCs) from TLNBLs in the ventricular zone, aiding biologists in further studying the dynamics of EGCs and the locally newly generated neurons (medial pallium excitatory neuron [mpEX]) during axolotl brain regeneration (Figure 5F).

Spaco is applicable to diverse technologies and tissue types

We extended our testing of Spaco to a seqFISH mouse embryo dataset[9] and an IMC human breast cancer dataset.[26] We sourced the color palettes for both datasets from the online tutorial of the Squidpy package.[17] The optimized color assignment and autogenerated palette by Spaco demonstrated superior visual clarity compared to other methods (Figures S4A and S4B). In the IMC dataset, Spaco's color assignment aligned with the manual assignment from the Squidpy tutorial, notably mapping cyan to the predominant apoptotic tumor cells. Furthermore, it excelled in differentiating between apoptotic and basal cytokeratins (CK) tumor cells located in the bottom right of the section (Figure S4B).

Spaco ensures broad accessibility by supporting diverse userships

As a color-based visualization method, Spaco was designed to adapt to various situations of CVD. Using an LMS (long, medium, short) space-based color conversion method,[32] Spaco was capable of simulating perceptual colors as perceived by individuals with CVD and modifying color difference calculations (see experimental procedures) to simultaneously consider three common types of CVD conditions: protanopia, deuteranopia, and tritanopia.

We integrated and tested this feature for both Spaco's color assignment (Figures S5A–S5D) and its image-guided palette extraction approach (Figures S5E–S5H) on the STARmap mouse brain dataset and the Stereo-seq axolotl dataset, respectively. We excluded our graph-embedding color selection from being CVD friendly due to the absence of a continuous, perceptually uniform CVD color space for UMAP embedding. Notably, Spaco's CVD-friendly colorization achieved improved discernibility compared visually to Scanpy's default colorization, not only in all three simulated CVD conditions (Figures S5B, S5D, and S5H) but also in full-color perception (Figures S5A, S5C, and S5G). This is important for ensuring broad readability of Spaco's visualization for diverse audiences in publications. In addition, Spaco also gives the option to perform CVD-friendly colorization under one given type of CVD, which intends to provide even better support for users with that CVD type in their daily work.

Another key aspect of our approach is to support user communities of both Python and R, the two predominant programming languages in the fields of single-cell and spatial omics. We have made available scripts related to all benchmarking experiments, which are particularly beneficial for users of Python and Scanpy. For the R community, we have constructed a tutorial that demonstrates the combined use of Spaco and Seurat, as illustrated in Figure S6. All of these code resources are available in open-access repositories, as detailed in the data and code availability section.

DISCUSSION

On one single spatial transcriptomics chip, millions of cells and hundreds of cell-type clusters organize in spatially structured arrangements. It is conceivable that the cell types can be either isolated, neighbored, partially interlaced, or completely mixed in a tissue, orchestrating the diverse microenvironmental niches. We therefore developed Spaco, an automatic palette selection and colorization tool, to solve this newly emerged data visualization problem. With Spaco, experienced biologists, anatomists, and pathologists can identify the pivotal cell clusters and quickly zoom in on the candidate biological interests based on their spatial characteristics, given their prior knowledge of tissue configuration.

As Spaco is a graph-based method, it possesses many computational applications rooted in graph structure, surpassing existing coloring algorithms. Notably, the identification of an appropriate edge metric to characterize the graph is in the first priority. To develop the DOI metric, we utilized the spatial k-nearest neighbor graph in the local neighborhoods of each cell, which serves as a discrete spatial kernel. Consequently, DOI is able to model the interlacement between spatially scattered cells in cellular SRT data rather than continuous domains in low-resolution SRTs. On the other hand, leveraging the interlacement graphs, Spaco offers the potential to assess variations in tissue topology across different sections by identifying changes in edges (i.e., DOIs). This capability could further facilitate the discovery of topologically conservative or variable cell types over timescales. These functionalities are not available in any other publicly available packages.

While Spaco effectively employs distinct colors to visualize the spatial organization of cell types for biological insights, other studies, such as Baysor[33] and Bento,[34] harness the color space for segmenting cells or even subcellular domains in spatial transcriptomic data analyses. The idea of utilizing color-based analyses to explore a variety of biological features is compelling. Another notable example is DeepVariant,[35] which introduced an approach where sequencing reads are encoded into RGB-colored images to enhance variant-calling accuracy. Though the majority of color space applications currently focus on data visualization, our attempts at downstream color-based graph analysis underscore the potential of adapting color-centric algorithms for broader biological research.

One major limitation of Spaco is its enforcement of a correspondence between color differences and the spatial distribution of cells. However, there might be scenarios where users wish to use similar colors for cell subtypes, regardless of their spatial relationships, or they might want to specify colors for certain cell types to align with existing literature. To address these situations, Spaco offers an option allowing users to manually fix or adjust colors for user-specified cell types and then optimize the color differences for the remaining cell types. Second, the visual contrast that can be obtained through color space is limited when performing CVD-supporting colorization or in the case of coloring hundreds of clusters. It would be helpful to exploit fine-designed CVD-supporting palettes[36,37] and more rich-formatted techniques like interactive plots and virtual reality. On a different note, while Spaco's algorithm is theoretically adept at processing 3D data, fine-tuning of parameters is crucial to differentiate "sparse" from "dense" cell types for efficient 3D colorization.

Spaco is implemented in Python using a modularized architecture to seamlessly integrate into a variety of analytical platforms and to incorporate emerging deep-learning-based clustering methods. This approach also facilitates community contributions, extension of new functions, and even the separation of

program functions to support individual scripts. As spatial technologies continue to evolve, potentially becoming the primary method for characterizing the molecular profiles of cells, we anticipate that Spaco will become an indispensable tool for researchers across a broad range of biological fields.

EXPERIMENTAL PROCEDURES

Resource availability

Lead contact

Further information and requests for resources and reagents should be directed to and will be fulfilled by the lead contact, Yinqi Bai (baiyinqi@genomics.cn).

Materials availability

This study did not generate new unique materials.

Data and code availability

All datasets are public and available for download from their original publications, which are listed as follows.

(1) The adult mouse brain STARmap dataset[24]: https://singlecell.broadinstitute.org/single_cell/study/SCP1830/.
(2) The wound-healing axolotl Stereo-seq datasets[25]: https://db.cngb.org/stomics/artista/download.
(3) The mouse embryo seqFISH dataset[9]: https://marionilab.cruk.cam.ac.uk/SpatialMouseAtlas/.
(4) The breast cancer IMC datasets[26]: https://doi.org/10.5281/zenodo.3518284.

Spaco is an open source, collaborative Python package available at Zenodo (https://zenodo.org/doi/10.5281/zenodo.10113347)[38] and also at the GitHub repository (https://github.com/BrainStOrmics/Spaco). The scripts to reproduce Spaco's results and figures are available at https://zenodo.org/doi/10.5281/zenodo.10113359[39] and https://github.com/BrainStOrmics/Spaco_scripts. For combinatorial use of Spaco with R, see https://github.com/BrainStOrmics/SpacoR.

Modeling spatial interlacement of clusters

Design of DOI

To characterize the spatial differentiation between clusters, we assess the interlacement of the cells within each cluster. One of the main challenges in calculating spatial interlacement by estimating cluster overlap in single-cell resolution spatial data arises when cells are sparsely distributed, resulting in variable density within the tissue section. This can lead to an overestimation of the spatial coverage of clusters.

To circumvent the risk of inferring incorrect interlacement relationships from such instances, we employ the k-dimensional tree algorithm to calculate k-nearest neighbors for all cells, with k defaulting to 30. This method draws inspiration from domain identification algorithms that utilize spatial neighbors.[17,40,41]

Additionally, we apply a dual-outlier-free strategy to refine this k-nearest neighbor calculation. The term "dual" pertains to two distinct outlier cell categories.

(1) The "sparse-type" outlier, characterized by having fewer than k cells within a specified radius r. This suggests that the cell occupies a sparsely populated region of the tissue.
(2) The "orphan-type" outlier, which lacks (or has fewer than a designated number n) cells of its cluster within its neighboring cells. This indicates that the cell might be isolated from the primary group of the cluster or that the cluster itself may have a sparse spatial configuration.

For sparse types, Spaco retains only the closest neighbors within the radius r (defaulting to 50 μm) to prevent incorrect associations with distant cells. For orphan types, we omit the orphan cells from the neighbor calculations, ensuring we do not overestimate the spatial coverage and interlacement of clusters.

Following these steps, the spatial interlacement is determined pairwise for all clusters using the refined cell-cell neighbor relationships. Let x and y denote the labels (indices) of two distinct clusters. We define the interlacement score between them, I_{xy}, as

$$I_{xy} = \sum_{i \in C_x} \frac{\sum_{j \in N_i, j \in C_y} \frac{1}{Dist(i,j)}}{|N_i|}$$

where C_x and C_y denote the cells in clusters x and y, respectively, and $|N_i|$ denotes the number of nearest neighbors of cell i. $Dist(i,j)$ is the Euclidean distance between cells i and j under the given spatial coordinates.

Constructing CI-graph

Basically, the interlacement score models the mean inversed distance between the neighboring cells of two clusters. Calculated scores are used to construct a weighted CI-graph, where vertices represent each cluster and edges are weighted by the interlacement scores (i.e., DOIs described in the previous part). The adjacency matrix of this graph is symmetrized (converted to undirected graph) by replacing I_{xy} and I_{yx} with $Max(I_{xy}, I_{yx})$ for any x and y, and the diagonal is set to zeros in the matrix.

Adaptive color palette selection

Generating palette using graph embedding

Spaco offers adaptive generation of a color palette by embedding the CI-graph into the CIELab color space.[29] To achieve this, we utilize UMAP[28] to compute a 3D embedding of all vertices in the CI-graph. In this configuration, less interlaced clusters present as hubs in the CI-graph since the DOIs (i.e., edge weights) among their corresponding vertices are relatively low. Consequently, these hubs are embedded into aggregately distributed points groups in the 3D UMAP space (like the case in Figure 4D), leading cluster pairs with lower DOIs to closer distances in the embedded space, and vice versa.

Subsequently, these embeddings are rescaled to fit the value range of the CIELab, a perceptually uniform color space where the difference between colors corresponds to the Euclidean distance. A similar method was adopted in previous studies to colorize single-molecule FISH (smFISH) data based on the niche composition of the captured molecules.[33]

In the CIELab color space, the L, a, and b values typically range within $[0, 100]$, $[-128, 127]$, and $[-128, 127]$, respectively. To prevent the generation of colors that are either too dark or too bright, we further limit the illuminant channel L to a range of $[20, 85]$ by default. The final step involves converting the Lab values into RGB hex values to suit general applications.

A limitation of using UMAP involves potential distortion in neighborhood and distance.[42] Nevertheless, this coarse alignment between DOIs and color differences was sufficient for colorization purposes, whose feasibility was proven in Baysor.[33] The non-linear nature of UMAP also contributes to Spaco's robustness against outliers (i.e., heavily isolated vertices in CI-graph causing polarized palettes selection). Optional linear embedding methods in Spaco, such as multidimensional scaling (MDS), can be used for outlier-free cases.

Spaco allows the direct utilization of the embedded colors as effective color mapping depending on the coarse correspondence between DOIs and color differences, and it also provides users options for further optimization in the cluster-color assignment, detailed in the final module.

Image-guided theme palette extraction

Spaco offers an additional feature for adaptive color palette selection: the extraction of theme colors from images, enabling users to oversee the palette selection process by designating preferred images. Drawing inspiration from recent advancements in computational visual media,[43] for a given image, we summarize the colors of all pixels in a given image under 3D CIELab color space, using a binning size of 20 (by default) on each dimension (channel). This aggregation condenses all raw pixel colors into a potential maximum of 8,000 distinct binned colors, each represented by the centroid color of its respective bin. Colors falling within the L (illumination channel) range of $[20, 85]$ by default (considered either too dark or excessively bright), as well as those occurring below the third percentile in frequency (considered non-theme colors), are excluded from further calculation.

The initial palette is progressively assembled from binned colors, selected in descending order of frequency until the desired number of colors is attained.

To prevent the selection of similar colors, we update frequencies during the process, employing the following equation[44]:

$$f_a^t = f_a^{t-1}\left(1 - exp\left(-\left(\frac{E(a,b)}{\sigma}\right)^2\right)\right)$$

where f_a^t denotes the frequency of color a after the t-th selection, with b denoting the pulled color. $E(a,b)$ is the CIEDE2000 color difference metric[45] in default. σ is set to 80 by default.

Once an initial palette is established, we initiate a refinement process for the palette. We iteratively replace each color in the palette using farthest point sampling[30] (FPS) to maximize the minimum distance between the replaced color and the rest of the palette, ensuring optimal linear separation. In each iteration, k (3 by default) colors that are farthest from the rest of the palette are selected. Subsequently, we evaluate the k potential replacements using the formula

$$S_a = w_s s_a + w_f f_a + d_a,$$

in which s_a represents the linear separation of color a and d_a represents the pre-calculated distance in FPS. w_s and w_f are weights to balance between s_a and f_a, defaulting to 0.15 and the inverse of 0.03% of the image's pixel number, respectively. The color with the highest score is then employed to replace the corresponding color in the current palette. The iteration process concludes when the coefficient of variation (CV) for all pairwise distances within the color palette surpasses the CV of edge weights in the CI-graph or when the number of iterations exceeds the pre-defined limit (set to 20 times the palette size by default). Finally, a conversion from Lab values to RGB hex values is executed to facilitate general usage.

Graph-based color assignment

Calculating perceptual differences and constructing CD-graph

Once an adequate color palette is available, we proceed to construct a CD-graph that captures the color relationships within the palette.

We quantify the pairwise differences between colors by employing a well-established weighted metric within the RGB color space[46]:

$$\bar{r} = \frac{1}{2}(R_1 + R_2)$$

$$\Delta C = \sqrt{\left(2+\frac{\bar{r}}{256}\right)\Delta R^2 + 4\Delta G^2 + \left(2+\frac{255-\bar{r}}{256}\right)\Delta B^2},$$

where R_1 and R_2 are the red channels of the compared colors. ΔC represents the perceptual difference between two RGB colors, and R, G, and B stand for the corresponding color channels. The computed differences are utilized as edge weights in the adjacency matrix of the CD-graph.

Graph matching via coarse-to-fine optimization

The primary objective of color assignment is to ensure that clusters containing closely located or intertwined cells are allocated colors with significant perceptual differences. By utilizing the adjacency matrices of our CI-graph and CD-graph, we can frame the solution to this color-mapping challenge as a graph-matching problem. In this problem, our aim is to establish a one-to-one correspondence between the vertices of the CI-graph and the CD-graph, thereby achieving optimal consistency between the edge weights of the two graphs.

Mathematically, we can express the optimization as an objective function $L(P)$ defined as

$$L(P) = \|PA_{CI-graph}P^T - A_{CD-graph}\|_F,$$

where P is a permutation matrix representing the bijective assignment, in which $P_{ij} \in \{0,1\}$ and $\sum_i P_{ij} = \sum_j P_{ij} = 1$. $A_{CI-graph}$ and $A_{CD-graph}$ are the weighted adjacency matrices of CI-graph and CD-graph. $\|M\|_F$ is the Frobenius norm of matrix M.

Then, we can define the optimal assignment P^* to be the solution of following optimization:

$$P^* = argmin_P\, L(P).$$

While graph-matching problems of this nature, closely related to the challenging quadratic assignment problem, are known to be NP-hard (NP, nondeterministic polynomial time),[47–49] in the context of cluster-color assignment, we anticipate that the number of clusters will be relatively modest, likely not exceeding thousands.

Therefore, similar to previous methodologies employed in the optimization of palettes for single-cell data,[22] we adopt a straightforward stochastic optimization approach employing a coarse-to-fine strategy. Specifically, Spaco initiates the process by generating a set of permutation matrices (default size of 2,000) and identifies the optimal permutation that yields the minimal loss within this set. Subsequently, Spaco randomly selects a pair of clusters and attempts to refine the loss by exchanging color assignments between the two clusters. This fine-tuning process is iterated until a maximum number of permutations is reached (default value of 5,000) or until the loss remains unchanged for 100 consecutive permutations.

Integrative colorization for multiple plots

Furthermore, Spaco extends its capabilities to encompass colorization across multiple datasets or label categories by seamlessly integrating multiple CI-graphs.

Prior to the integration of CI-graphs, it is imperative to establish correspondences among identical cell labels present across distinct datasets. Spaco achieves this by evaluating gene expression similarity between clusters from different datasets, utilizing Pearson correlation across the top 5,000 highly variable genes. This similarity is denoted as $S'_{n\times m}$, where n and m are the numbers of unique clusters in two different plots.

Similarly, when aligning distinct label categories, such as when tuning the resolution in the clustering refinement process, the intersection over union (IoU) is employed in place of expression similarities.

Once S' is calculated, we employ a greedy approach to iteratively map i' and j', where

$$i', j' = argmax_{i,j}\, S'_{i,j}.$$

The corresponding row and column of the mapped indices in S' are then set to zero to ensure injective mapping. This iteration continues until all clusters have been mapped or until $max_{i,j}\, S'_{i,j}$ falls below a certain threshold (default to 0 for IoU similarity and 0.5 for normalized expression similarity). Unmapped clusters remain distinct after the iteration concludes. Subsequently, cluster labels in various plots are transformed into unified labels using the established mapping, facilitating the integration of CI-graphs.

CVD support

When performing a colorblind-friendly colorization, Spaco slightly adjusts the calculation relating to color differences. Colors are converted to three common types of CVD conditions including protanopia, deuteranopia, and tritanopia, following a previously established transformation.[50] Then, color perceptual differences are calculated separately using these three sets of simulated CVD colors. Finally, the mean value of these three CVD-simulating color differences and a normally calculated full-color-vision color difference is used (1) in the cluster-color assignment module, as edge weights in the adjacency matrix of the CD-graph, and (2) in the image-palette-extraction module, as color differences to punish color frequency. As for colorblind support for one certain type of CVD, only color differences under the given type of CVD simulation are used.

Benchmarking on simulated data

Generation of simulated dataset

In order to comprehensively evaluate the distance calculation methods for the DOI (Spaco), Wasserstein distance, LISI, and 2D KDE (Palo), we utilized the "normal" function from the "*numpy.random*" module to generate multiple sets of 2D points with x and y coordinates following a normal distribution with a standard deviation of 25. These point sets were used to simulate spatial topological relationships. We selected two sets of points with center-to-center distances of 10, 50, 70, 120, and 300, representing five spatial topologies: mixed, overlapped, adjacent, isolated, and distant. Specifically, we

generated two types of point sets: dense and sparse. Each dense point set contained 100 points, while each sparse point set contained 25 points. We assessed the results of different calculation methods under the aforementioned five spatial topologies in three scenarios: dense point set with dense point set, sparse point set with sparse point set, and dense point set with sparse point set.

Calculation of Wasserstein distance

We utilized the Python Optimal Transport (POT; https://pythonot.github.io/index.html) package to compute the Wasserstein distance between pairwise point sets. The POT package offers several solvers related to Optimal Transport. Specifically, we used the "*dist*" function provided in the package to calculate the distance matrix between individual points of the two sets. Subsequently, we used the "emd2" function to solve the Earth Mover's distance problem, where the weights of each point were set to be equal. The resulting loss obtained from this computation is considered as the Wasserstein distance between the two point sets.

Calculation of LISI

The LISI represents the average number of labels present in the N-nearest points.[21] We used the "*lisi*" function provided in the "scPOP"' R package (https://github.com/vinay-swamy/scPOP) to calculate the LISI between two sets of points. Specifically, we inputted all the points from both sets into the function, using the categories of each point as the label. The remaining parameters required by function *lisi* were set to their default values. The resulting LISI value between the two point sets was determined by averaging the LISI values of individual points. To invoke the functionality of R packages in Python, we utilized the "rpy2" package (https://github.com/rpy2/rpy2).

Calculation of 2D KDE

The Palo method provides another approach for the color assignment of palettes considering the spatial relationships of single-cell data.[22] The Palo method consists of three components: spatial overlap score calculation, color dissimilarities calculation, and color assignments. All functionalities are encompassed within the *Palo* function provided in the R package Palo. To meet our evaluation requirements, we extracted the functionality for calculating spatial overlap scores between point sets from the Palo function. We used our complete set of points and categories information as input, and the spatial overlap scores between the point sets calculated by the Palo function were considered as the distances of sets.

Benchmarking on real-world data

Dataset collection

To evaluate the efficacy of Spaco, we collected four distinct real-world datasets spanning different technologies and tissue types.[9,24–26] Each of these datasets has been previously analyzed, as detailed in their original publications. They provide crucial information for Spaco, including expression data, spatial coordinates, and labeling details. These labels represent cell types and anatomical annotations for individual cells or spatial spots. All datasets are publicly available and can be obtained directly from their source publications.

Measuring perceptual discernibility

As most colors are presented in hexadecimal form of RGB color space, we use the same metric as in CD-graph construction (see experimental procedures: calculating perceptual differences) and employ a CVD simulation (see experimental procedures: CVD support) when benchmarking CVD-supporting colorizations. We measure the discernibility between cell clusters as the perceptual difference between the corresponding colors. For micro-FOVs, the discernibility of a FOV is evaluated as the mean color difference of all cell pairs derived in this FOV. For palettes, the discernibility of a palette is measured as the mean color difference of all color pairs derived in the palette.

Blind user scoring of colorized spatial plots

To supplementarily assess Spaco's colorization performance from a user-centric viewpoint, we conducted a single-blind experiment. This experiment involved the creation of a questionnaire administered to 53 participants, all of whom possessed expertise in spatial transcriptomics analysis. The experiment aimed to evaluate the colorization of four distinct plots derived from the STARmap mouse brain dataset[24] using Spaco, Scanpy (matplotlib), random shuffling, and Palo.

Participants were requested to judge the discernibility of all cell clusters in each plot. To provide a reference of cluster distributions, the questionnaire was supplemented with split plots that highlighted each individual cell cluster. Participants were asked to assign scores to these colorized plots, rating them on a scale from 1 (worst) to 10 (best), based on their perceived quality. The original questionnaire and the gathered results are accessible through the following link: https://github.com/BrainStOrmics/Spaco_scripts.

This experimental design allows us to comprehensively gauge and compare the effectiveness of Spaco's colorization with other methods and to draw insightful conclusions from the gathered evaluations. However, a lack of participants with CVD made this survey limited to a full-color-vision perspective. Also, this survey should only be interpreted as a supplementary evaluation paratactic to our computational metric and did not lead to statistical conclusions due to insufficient sample size and potential bias of individual scoring criteria.

SUPPLEMENTAL INFORMATION

Supplemental information can be found online at https://doi.org/10.1016/j.patter.2023.100915.

ACKNOWLEDGMENTS

This work was supported by the National Key R&D Program of China (2022YFC3400400) (X.X.) and the Guangdong Provincial Key Laboratory of Genome Read and Write (X.X.). We are sincerely thankful for the technical support provided by China National Gene Bank.

AUTHOR CONTRIBUTIONS

Conceptualization, Y.B. and H.P.; methodology, Z.J. and Q.Z.; software, Z.J., Q.Z., and L.L.; formal analysis, Z.J., L.L., Y.X., and Q.Z.; validation, Z.J. and Y.X.; investigation, Y.X. and B.Y.; writing – original draft, Z.J.; writing – review & editing, Z.J., Y.B., X.W., L.L., H.P., Y.X., Q.F., and B.D.; funding acquisition, X.X.; resources, Y.B. and X.X.; supervision, Y.B., H.P., and X.X.

DECLARATION OF INTERESTS

The authors declare no competing interests.

DECLARATION OF GENERATIVE AI AND AI-ASSISTED TECHNOLOGIES IN THE WRITING PROCESS

During the preparation of this work, the authors used ChatGPT 4.0 (GPT-4) in order to facilitate the process of proofreading the contents of our draft. After using this tool/service, the authors reviewed and edited the content as needed and take full responsibility for the content of the publication.

Received: September 28, 2023
Revised: December 11, 2023
Accepted: December 18, 2023
Published: January 16, 2024

REFERENCES

1. Rao, A., Barkley, D., França, G.S., and Yanai, I. (2021). Exploring tissue architecture using spatial transcriptomics. Nature *596*, 211–220.
2. Wang, X., Allen, W.E., Wright, M.A., Sylwestrak, E.L., Samusik, N., Vesuna, S., Evans, K., Liu, C., Ramakrishnan, C., Liu, J., et al. (2018). Three-dimensional intact-tissue sequencing of single-cell transcriptional states. Science *361*, eaat5691.
3. Eng, C.-H.L., Lawson, M., Zhu, Q., Dries, R., Koulena, N., Takei, Y., Yun, J., Cronin, C., Karp, C., Yuan, G.-C., and Cai, L. (2019). Transcriptome-scale super-resolved imaging in tissues by RNA seqFISH. Nature *568*, 235–239.
4. Ståhl, P.L., Salmén, F., Vickovic, S., Lundmark, A., Navarro, J.F., Magnusson, J., Giacomello, S., Asp, M., Westholm, J.O., Huss, M., et al. (2016). Visualization and analysis of gene expression in tissue sections by spatial transcriptomics. Science *353*, 78–82.

5. Chen, A., Liao, S., Cheng, M., Ma, K., Wu, L., Lai, Y., Qiu, X., Yang, J., Xu, J., Hao, S., et al. (2022). Spatiotemporal transcriptomic atlas of mouse organogenesis using DNA nanoball-patterned arrays. Cell *185*, 1777–1792.e21.
6. Chen, A., Sun, Y., Lei, Y., Li, C., Liao, S., Meng, J., Bai, Y., Liu, Z., Liang, Z., Zhu, Z., et al. (2023). Single-cell spatial transcriptome reveals cell-type organization in the macaque cortex. Cell *186*, 3726–3743.e24.
7. La Manno, G., Siletti, K., Furlan, A., Gyllborg, D., Vinsland, E., Mossi Albiach, A., Mattsson Langseth, C., Khven, I., Lederer, A.R., Dratva, L.M., et al. (2021). Molecular architecture of the developing mouse brain. Nature *596*, 92–96.
8. Zeng, B., Liu, Z., Lu, Y., Zhong, S., Qin, S., Huang, L., Zeng, Y., Li, Z., Dong, H., Shi, Y., et al. (2023). The single-cell and spatial transcriptional landscape of human gastrulation and early brain development. Cell Stem Cell *30*, 851–866.e7.
9. Lohoff, T., Ghazanfar, S., Missarova, A., Koulena, N., Pierson, N., Griffiths, J.A., Bardot, E.S., Eng, C.-H.L., Tyser, R.C.V., Argelaguet, R., et al. (2022). Integration of spatial and single-cell transcriptomic data elucidates mouse organogenesis. Nat. Biotechnol. *40*, 74–85.
10. Berglund, E., Maaskola, J., Schultz, N., Friedrich, S., Marklund, M., Bergenstråhle, J., Tarish, F., Tanoglidi, A., Vickovic, S., Larsson, L., et al. (2018). Spatial maps of prostate cancer transcriptomes reveal an unexplored landscape of heterogeneity. Nat. Commun. *9*, 2419.
11. Wu, L., Yan, J., Bai, Y., Chen, F., Zou, X., Xu, J., Huang, A., Hou, L., Zhong, Y., Jing, Z., et al. (2023). An invasive zone in human liver cancer identified by Stereo-seq promotes hepatocyte-tumor cell crosstalk, local immunosuppression and tumor progression. Cell Res. *33*, 585–603.
12. Liu, B., Li, Y., and Zhang, L. (2021). Analysis and Visualization of Spatial Transcriptomic Data. Front. Genet. *12*, 785290.
13. Crameri, F., Shephard, G.E., and Heron, P.J. (2020). The misuse of colour in science communication. Nat. Commun. *11*, 5444.
14. Butler, A., Hoffman, P., Smibert, P., Papalexi, E., and Satija, R. (2018). Integrating single-cell transcriptomic data across different conditions, technologies, and species. Nat. Biotechnol. *36*, 411–420.
15. Dries, R., Zhu, Q., Dong, R., Eng, C.-H.L., Li, H., Liu, K., Fu, Y., Zhao, T., Sarkar, A., Bao, F., et al. (2021). Giotto: a toolbox for integrative analysis and visualization of spatial expression data. Genome Biol. *22*, 78.
16. Wolf, F.A., Angerer, P., and Theis, F.J. (2018). SCANPY: large-scale single-cell gene expression data analysis. Genome Biol. *19*, 15.
17. Palla, G., Spitzer, H., Klein, M., Fischer, D., Schaar, A.C., Kuemmerle, L.B., Rybakov, S., Ibarra, I.L., Holmberg, O., Virshup, I., et al. (2022). Squidpy: a scalable framework for spatial omics analysis. Nat. Methods *19*, 171–178.
18. Hunter, J.D. (2007). Matplotlib: A 2D Graphics Environment. Comput. Sci. Eng. *9*, 90–95.
19. Wickham, H. (2011). Ggplot2. WIREs Computational Stats. *3*, 180–185.
20. Vaserstein, L.N. (1969). Markov processes over denumerable products of spaces, describing large systems of automata. Probl. Peredachi Inf. *5*, 64–72.
21. Korsunsky, I., Millard, N., Fan, J., Slowikowski, K., Zhang, F., Wei, K., Baglaenko, Y., Brenner, M., Loh, P.-R., and Raychaudhuri, S. (2019). Fast, sensitive and accurate integration of single-cell data with Harmony. Nat. Methods *16*, 1289–1296.
22. Hou, W., and Ji, Z. (2022). Palo: spatially aware color palette optimization for single-cell and spatial data. Bioinformatics *38*, 3654–3656.
23. Neuwirth, E., and Brewer, R.C. (2014). Package RColorBrewer palettes. ColorBrewer palettes, 1.
24. Shi, H., He, Y., Zhou, Y., Huang, J., Maher, K., Wang, B., Tang, Z., Luo, S., Tan, P., Wu, M., et al. (2023). Spatial atlas of the mouse central nervous system at molecular resolution. Nature *1–10*.
25. Wei, X., Fu, S., Li, H., Liu, Y., Wang, S., Feng, W., Yang, Y., Liu, X., Zeng, Y.-Y., Cheng, M., et al. (2022). Single-cell Stereo-seq reveals induced progenitor cells involved in axolotl brain regeneration. Science *377*, eabp9444.
26. Jackson, H.W., Fischer, J.R., Zanotelli, V.R.T., Ali, H.R., Mechera, R., Soysal, S.D., Moch, H., Muenst, S., Varga, Z., Weber, W.P., and Bodenmiller, B. (2020). The single-cell pathology landscape of breast cancer. Nature *578*, 615–620.
27. Virshup, I., Rybakov, S., Theis, F.J., Angerer, P., and Alexander Wolf, F. (2021). anndata: Annotated data. Preprint at bioRxiv. https://doi.org/10.1101/2021.12.16.473007.
28. McInnes, L., Healy, J., and Melville, J. (2018). UMAP: Uniform Manifold Approximation and Projection for Dimension Reduction. Preprint at arXiv. https://doi.org/10.48550/arXiv.1802.03426.
29. Zhang, X., and Wandell, B.A. (2012). A Spatial Extension of CIELAB for digital color image reproduction. J. Soc. Inf. Display *5*, 61–63.
30. Eldar, Y., Lindenbaum, M., Porat, M., and Zeevi, Y.Y. (1997). The farthest point strategy for progressive image sampling. IEEE Trans. Image Process. *6*, 1305–1315.
31. Sunkin, S.M., Ng, L., Lau, C., Dolbeare, T., Gilbert, T.L., Thompson, C.L., Hawrylycz, M., and Dang, C. (2013). Allen Brain Atlas: an integrated spatio-temporal portal for exploring the central nervous system. Nucleic Acids Res. *41*, D996–D1008.
32. Brettel, H., Viénot, F., and Mollon, J.D. (1997). Computerized simulation of color appearance for dichromats. J. Opt. Soc. Am. Opt Image Sci. Vis. *14*, 2647–2655.
33. Petukhov, V., Xu, R.J., Soldatov, R.A., Cadinu, P., Khodosevich, K., Moffitt, J.R., and Kharchenko, P.V. (2022). Cell segmentation in imaging-based spatial transcriptomics. Nat. Biotechnol. *40*, 345–354.
34. Mah, C.K., Ahmed, N., Lam, D., Monell, A., Kern, C., Han, Y., Cesnik, A.J., Lundberg, E., Zhu, Q., Carter, H., et al. (2022). Bento: A toolkit for subcellular analysis of spatial transcriptomics data. Preprint at bioRxiv. https://doi.org/10.1101/2022.06.10.495510.
35. Poplin, R., Chang, P.-C., Alexander, D., Schwartz, S., Colthurst, T., Ku, A., Newburger, D., Dijamco, J., Nguyen, N., Afshar, P.T., et al. (2018). A universal SNP and small-indel variant caller using deep neural networks. Nat. Biotechnol. *36*, 983–987.
36. Bunis, D.G., Andrews, J., Fragiadakis, G.K., Burt, T.D., and Sirota, M. (2021). dittoSeq: universal user-friendly single-cell and bulk RNA sequencing visualization toolkit. Bioinformatics *36*, 5535–5536.
37. Crameri, F. (2023). Scientific Colour Maps (8.0.1). Zenodo. https://doi.org/10.5281/zenodo.1243862.
38. Jing, Z., and Zhu, Q. (2023). BrainStOrmics/Spaco: Spaco 0.2. Zenodo. https://doi.org/10.5281/zenodo.10121765.
39. Jing, Z. (2023). BrainStOrmics/Spaco_scripts: Spaco_scripts_231111. Zenodo. https://doi.org/10.5281/zenodo.10113360.
40. Zhao, E., Stone, M.R., Ren, X., Guenthoer, J., Smythe, K.S., Pulliam, T., Williams, S.R., Uytingco, C.R., Taylor, S.E.B., Nghiem, P., et al. (2021). Spatial transcriptomics at subspot resolution with BayesSpace. Nat. Biotechnol. *39*, 1375–1384.
41. Hu, J., Li, X., Coleman, K., Schroeder, A., Ma, N., Irwin, D.J., Lee, E.B., Shinohara, R.T., and Li, M. (2021). SpaGCN: Integrating gene expression, spatial location and histology to identify spatial domains and spatially variable genes by graph convolutional network. Nat. Methods *18*, 1342–1351.
42. Chari, T., and Pachter, L. (2023). The specious art of single-cell genomics. PLoS Comput. Biol. *19*, e1011288.
43. Zheng, Q., Lu, M., Wu, S., Hu, R., Lanir, J., and Huang, H. (2022). Image-guided color mapping for categorical data visualization. Comput. Vis. Media (Beijing). *8*, 613–629.
44. Zhang, Q., Xiao, C., Sun, H., and Tang, F. (2017). Palette-Based Image Recoloring Using Color Decomposition Optimization. IEEE Trans. Image Process. *26*, 1952–1964.

CellPress
OPEN ACCESS

45. Luo, M.R., Cui, G., and Rigg, B. (2001). The development of the CIE 2000 colour-difference formula: CIEDE2000. Color Res. Appl. *26*, 340–350.

46. Riemersma, T. (2019). Colour Metric. https://www.compuphase.com/cmetric.htm.

47. Grohe, M., Rattan, G., and Woeginger, G.J. (2018). Graph Similarity and Approximate Isomorphism. Preprint at arXiv. https://doi.org/10.48550/arXiv.1802.08509.

48. Koopmans, T.C., and Beckmann, M. (1957). Assignment problems and the location of economic activities. Econometrica *25*, 53.

49. Sahni, S., and Gonzalez, T. (1976). P-complete approximation problems. J. ACM *23*, 555–565.

50. Elrefaei, L.A. (2018). Smartphone Based Image Color Correction for Color Blindness. Int. J. Interact. Mob. Technol. *12*, 104–119.

Patterns, Volume *5*

Supplemental information

Spaco: A comprehensive tool for coloring spatial data at single-cell resolution

Zehua Jing, Qianhua Zhu, Linxuan Li, Yue Xie, Xinchao Wu, Qi Fang, Bolin Yang, Baojun Dai, Xun Xu, Hailin Pan, and Yinqi Bai

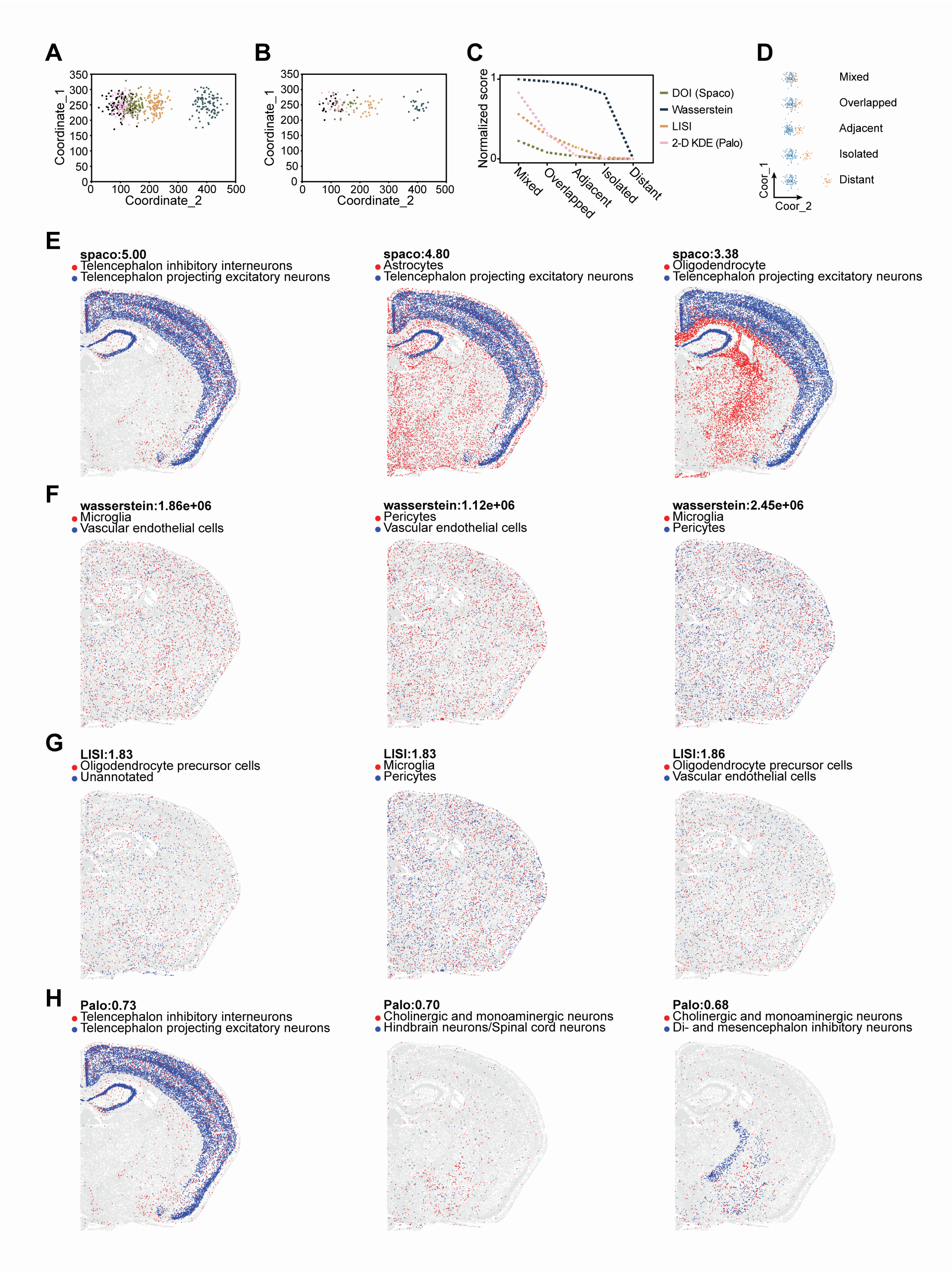

Supplementary Data Figure 1. Evaluation of spatial relation metrics on STARmap mouse brain data

(A) Five groups of points generated to simulate cell type distributions in SRT datasets.

(B) Parallel to panel **A**, with points generated to represent a sparse distribution (see **Methods**).

(C) Min-max normalized scores of five differently distributed cluster pairs in simulation data, evaluated using various metrics. This is akin to **Figure 2B** and **Figure 2C**, but the cluster pairs here are chosen between dense and sparse clusters.

(D) Visualization detailing the spatial distribution of each cluster pair evaluated in panel **C**.

(E-H) Top three scored cluster pairs in the STARmap mouse brain dataset as determined by the DOI metric (**E**), Wasserstein distance (**F**), LISI (**G**) and 2-D KDE-Jaccard index (**H**). The scores and normalized scores (enclosed in brackets) are labeled for each cluster pair and metric.

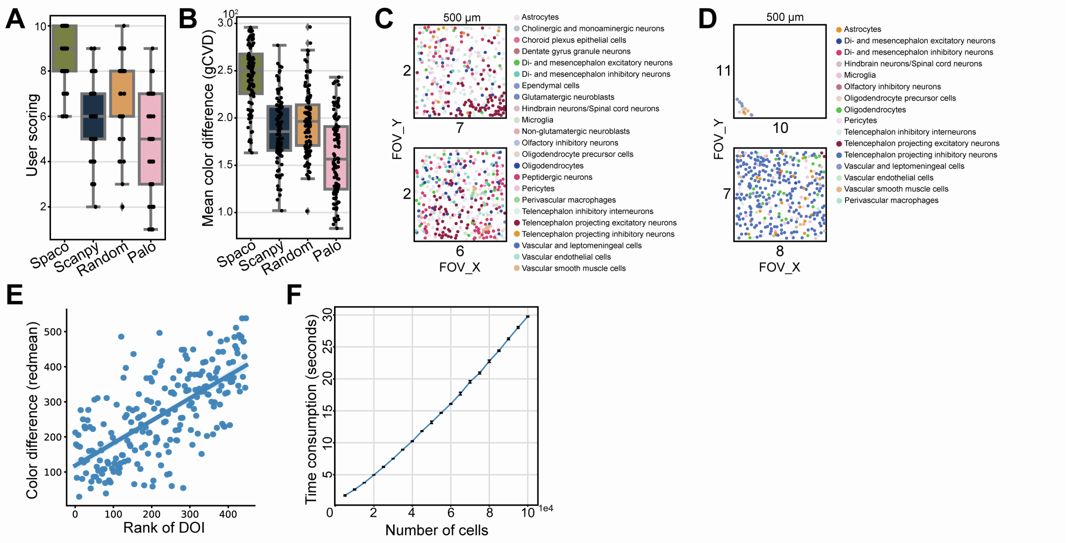

Supplementary Data Figure 2. Evaluation of cluster interlacement scoring and cluster-color matching in Spaco

(A) Blind user scoring of the visualizations from different methods.

(B) Same as **Figure 3F**, but for Spaco's general-CVD support mode.

(C) Same as **Figure 3G**, but for Spaco's general-CVD support mode.

(D) Spaco visualization of the two FOVs with lowest color difference, similar to **Figure 3G**.

(E) Consistency between interlacement scoring and color difference optimized by Spaco, demonstrated by linear regression.

(F) Time consumption of Spaco's color assignment across varying data scales, ranging from 5,000 to 100,000 cells; error bars represent standard deviation.

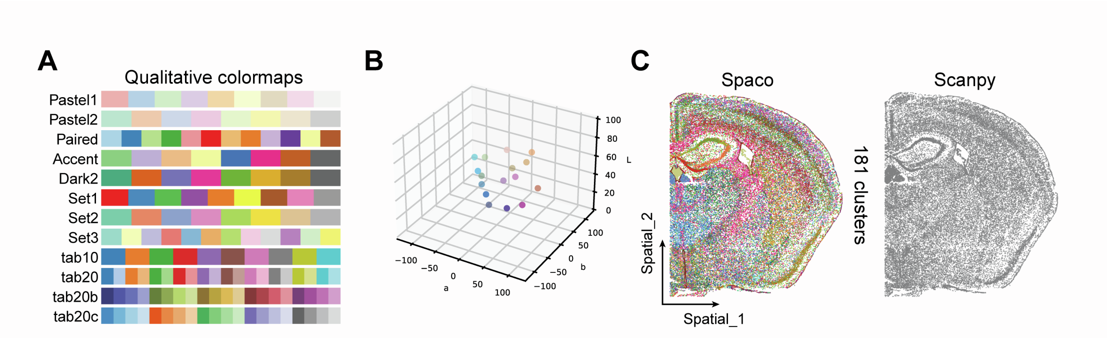

Supplementary Data Figure 3. Evaluation of adaptive graph-guided and image-guided palette selection in Spaco

(A) Commonly used predefined palettes.

(B) Visualization of the colors from the Spaco-generated palette in the 3D CIELab space.

(C) Colorization of 181 cell types annotated in the STARmap dataset using an auto-generated palette by Spaco's cluster interlacement graph embedding (**left**), compared to the glitched palette in Scanpy's default plot (**right**).

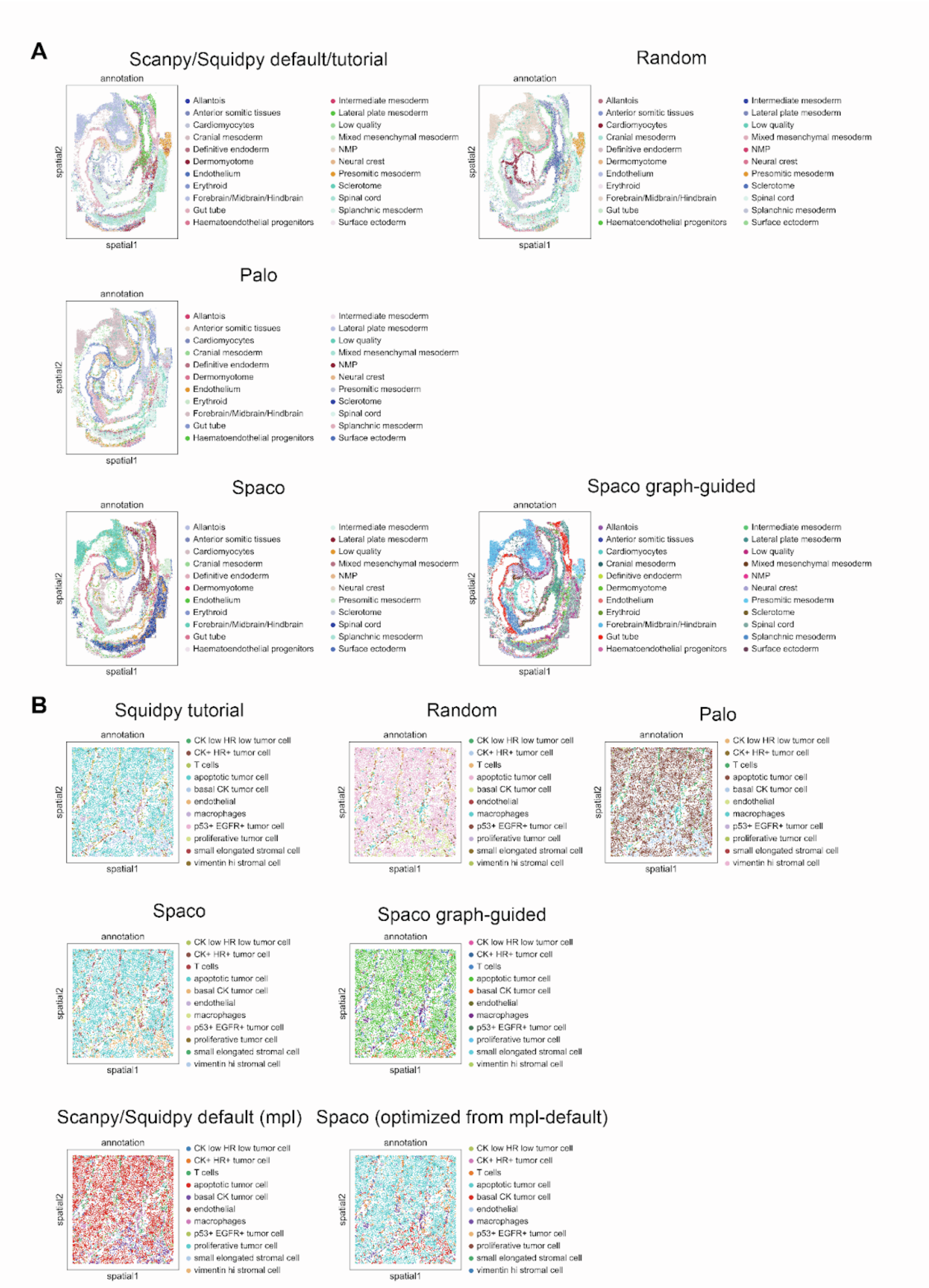

Supplementary Data Figure 4. Benchmarking Spaco on seqFISH and IMC datasets

(A) Colorization of the seqFISH dataset using various colorization strategies. All plots, with the exception of the Spaco graph-guided plot, use the same color palette as the Scanpy/Squidpy default plot, which is also featured in Squidpy tutorials.

(B) Colorization of the IMC dataset using different colorization strategies. The first four plots utilize the color palette from the Squidpy tutorial, while the subsequent plots adopt

the color palette from the Scanpy/Squidpy default plot.

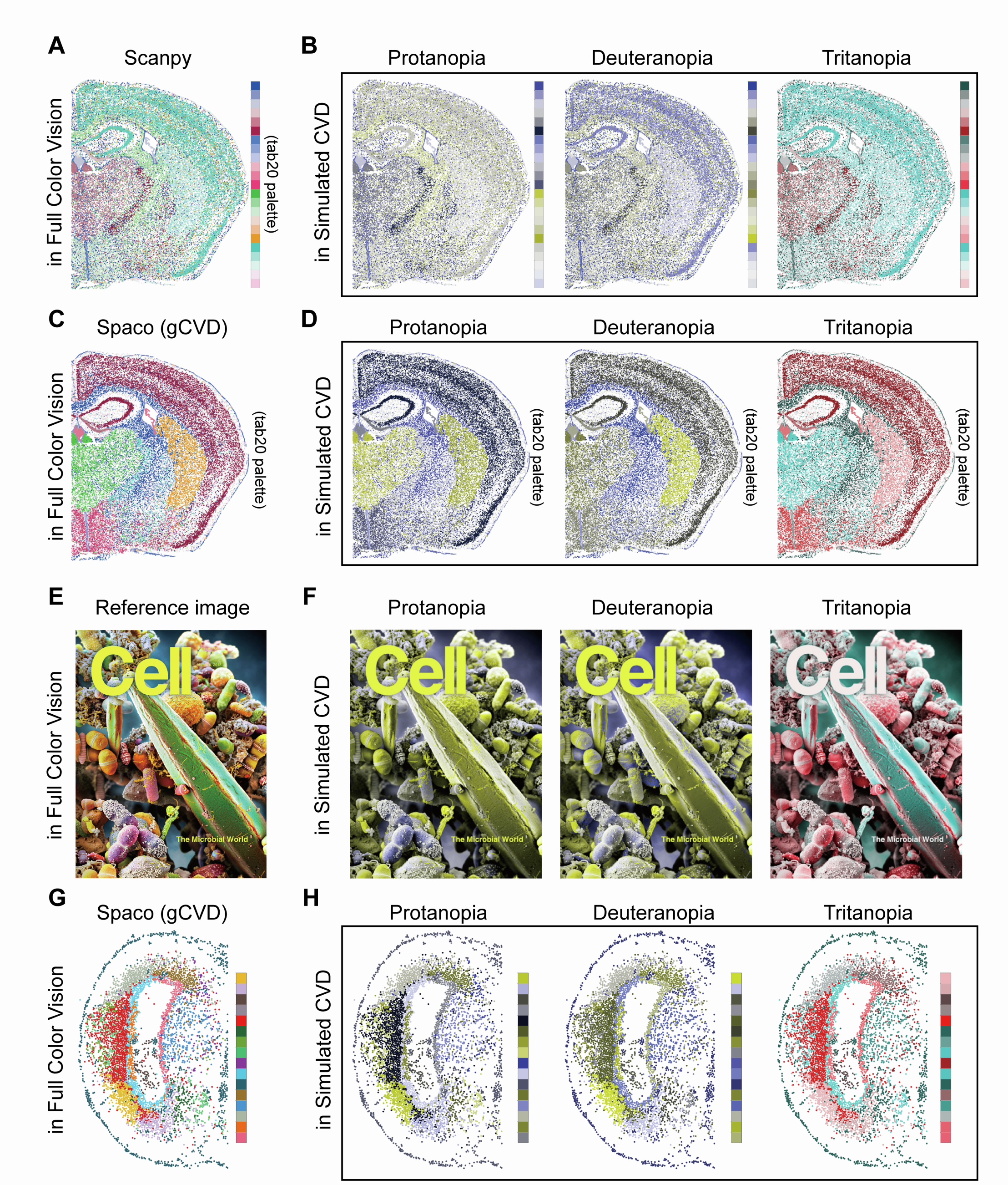

Supplementary Data Figure 5. Testing Spaco's performance for CVD-friendly colorization

(A) Scanpy's default visualization of STARmap mouse brain dataset, demonstrated with full color perception.

(B) Perceptual simulation of different CVDs of Scanpy's default visualization.

(C) Spaco's optimized color assignment of STARmap mouse brain dataset using general-CVD-friendly mode, demonstrated with full color perception.

(D) Same as panel **C**, but plotted with three types of CVD perceptual simulation.

(E) Reference image used in Spaco's palette extraction. Visualized in full color perception.

(F) Same as panel **E**, but visualized in simulated CVD color perception.

(G) Spaco's general-CVD-friendly-mode colorization with image-guided palette extraction on Stereo-seq axolotl brain dataset. Plotted with full color perception.

(H) Same as panel **G**, but plotted to simulate CVD color perception.

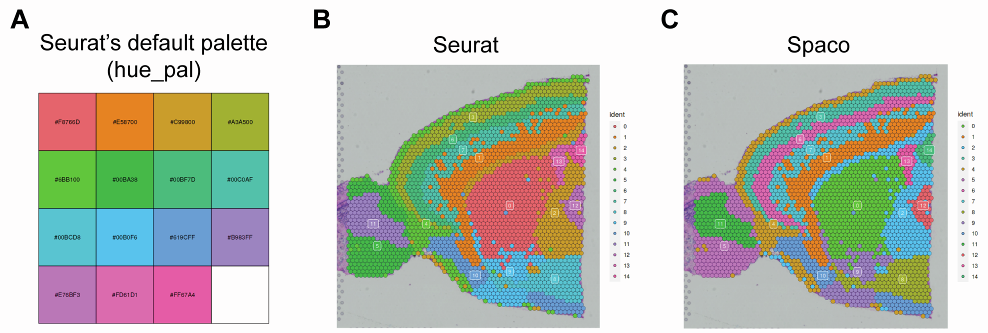

Supplementary Data Figure 6. Demonstration of Spaco’s collaboration with Seurat

(A) Seurat’s default palette selection on 10X Visium mouse brain dataset.

(B) Seurat’s default visualization of 10X Visium mouse brain dataset.

(C) Seurat’s default visualization of 10X Visium mouse brain dataset using optimized cluster-color assignment.
